# Supplementary material for: Magnesium in acute pediatric asthma in the emergency department (MAGICIAN)—A multicentre randomized controlled clinical trial protocol
Source: PLoS One. 2026 Jun 3;21(6):e0349553. doi: 10.1371/journal.pone.0349553 (PMC13232952; doi:10.1371/journal.pone.0349553)
Supplement: S1 File — (DOCX) [file pone.0349553.s001.docx]

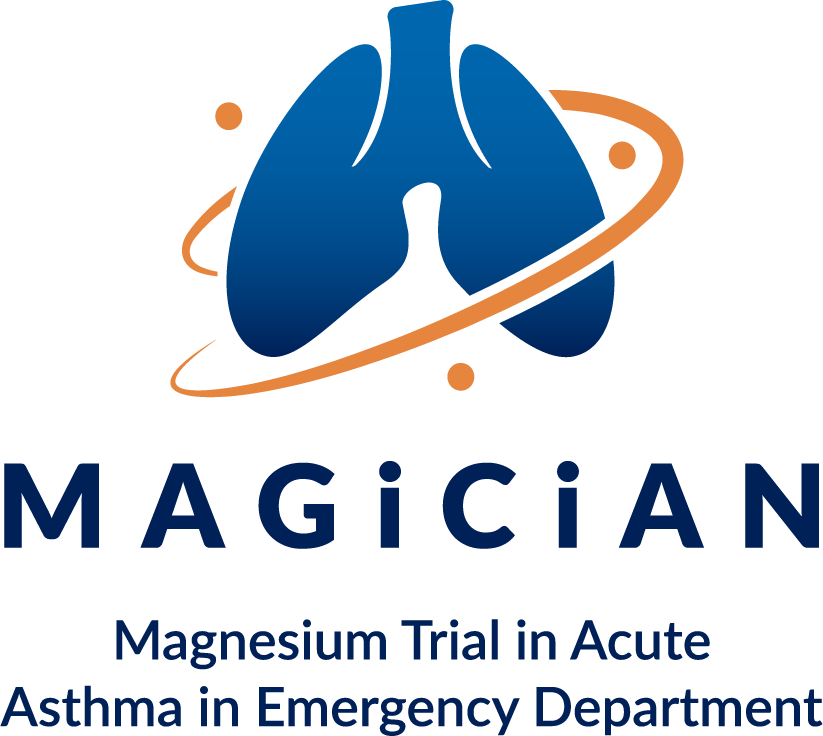


Version Date: June 25, 2025 (v3.0)

Page **1** of **54**


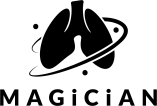


**Collaborators**

Dr. Yaron Finkelstein, The Hospital for Sick Children, Toronto, Ontario (Co-PI Grant) Dr. Allan Coates, The Hospital for Sick Children, Toronto, Ontario

Dr. Roger Zemek, Children’s Hospital of Eastern Ontario

Mark Bedford, The Hospital for Sick Children, Toronto, Ontario Dr. Francine Ducharme, CHU Ste-Justine, Montreal, Quebec

Dr Stephen Freedman, Alberta Children’s Hospital, Calgary, Alberta

Dr. Terry Klassen, University of Saskatchewan, Saskatoon, Saskatchewan Dr. George Wells, University of Ottawa Heart Institute, Ottawa, Ontario Dr. Jesse Elliot, University of Ottawa, Ontario

**Protocol Registration:** Clinicaltrials.gov registration number: NCT06785272

**MAGICIAN** Clinical Trial Protocol

**Study Title:** Magnesium Trial in Acute Asthma in Emergency Department

**Short Title:** MAGICIAN

**Protocol Number:** CTO 5274

**Protocol Version #: 3.0**

**Protocol Date: June 25, 2025**

**Phase of Study: Phase III**

**Sponsor:** The Hospital for Sick Children

**Study Funder:** Canadian Institutes of Health Research (CIHR)

**Study Chair (Sponsor-Investigator):** Suzanne Schuh MD

**Site Principal Investigators (Qualified Investigators):**

Suzanne Schuh, MD Staff Physician

555 University Avenue Hospital for Sick Children Toronto, Canada. M5G 1X8

Email: [suzanne.schuh@sickkids.ca](mailto:suzanne.schuh@sickkids.ca) Phone: 416-819-7654 x406239

April Kam, MD

Pediatric Emergency Physician McMaster University

1280 Main Street W Hamilton, Canada. L8S 4L8

Waleed Alqurashi, MD Pediatric Emergency Physician 401 Smyth Rd

Children’s Hospital of Eastern Ontario (CHEO)

Ottawa, Canada. K1H 8L1

Mohamed Eltorki, MD Pediatric Emergency Physician Alberta Children’s Hospital

28 Oki Drive NW

Calgary, Canada. T3B 6A8

Jocelyn Gravel, MD

Pediatric Emergency Physician CHU Sainte-Justine

3175 Chem. de la Côte-Sainte-Catherine Montreal Quebec. H3T 1C5

Sarah Curtis, MD

Pediatric Emergency Physician Stollery Children’s Hospital 8440 112 St NW

Edmonton, AB Canada. T6G 2B7

Version Date: June 25, 2025 (v3.0)

Page **2** of **54**


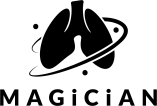


**MAGICIAN** Clinical Trial Protocol

Table of Contents

[STATEMENT OF COMPLIANCE 6](#_bookmark0)

[1.](#_bookmark1)

[1.1.](#_bookmark2)

[1.2.](#_bookmark3)

[1.3.](#_bookmark4)

[2.](#_bookmark5)

[2.1.](#_bookmark6)

[2.2.](#_bookmark7)

[2.3.](#_bookmark8)

[3.](#_bookmark9)

[4.](#_bookmark10)

[4.1.](#_bookmark11)

[4.2.](#_bookmark12)

[5.](#_bookmark13)

[5.1.](#_bookmark14)

[5.2.](#_bookmark15)

[5.3.](#_bookmark16)

[6.](#_bookmark17)

[6.1.](#_bookmark18)

[6.2.](#_bookmark19)

[6.3.](#_bookmark20)

[6.4.](#_bookmark21)

[PROTOCOL SUMMARY 7](#_bookmark1)

[Synopsis 7](#_bookmark2)

[Schema 9](#_bookmark3)

[Schedule of Activities (SoA) 10](#_bookmark4)

[INTRODUCTION 11](#_bookmark5)

[Study Rationale - The Need For A Trial 11](#_bookmark6)

[Background 12](#_bookmark7)

[Risk/Benefit Assessment - Participant Safety 13](#_bookmark8)

[OBJECTIVES AND END POINTS 14](#_bookmark9)

[STUDY DESIGN 15](#_bookmark10)

[Overall Design and the Scientific Rationale for Study Design 15](#_bookmark11)

[End of Study Definition 15](#_bookmark12)

[STUDY POPULATION 16](#_bookmark13)

[Inclusion criteria 16](#_bookmark14)

[Exclusion criteria: 16](#_bookmark15)

[Strategies For Recruitment and Retention 16](#_bookmark16)

[STUDY INTERVENTION 17](#_bookmark17)

[Study Intervention Description 17](#_bookmark18)

[Study procedures (Dosing and Administration) 17](#_bookmark19)

[Study Procedures (after Administration of Study Intervention) 18](#_bookmark20)

[Preparation/Handling/Storage/Accountability 19](#_bookmark21)

[6.4.1.](#_bookmark22)

[6.4.2.](#_bookmark23)

[6.4.3.](#_bookmark24)

[6.5.](#_bookmark25)

[6.6.](#_bookmark26)

[6.7.](#_bookmark27)

[Acquisition and Accountability 19](#_bookmark22)

[Preparation of Treatment allocation and storage 19](#_bookmark23)

[Logistics of Blinding and Kit Making 19](#_bookmark24)

[Measures to Minimize Bias: Randomization and Blinding 20](#_bookmark25)

[Emergency Unblinding Procedures 21](#_bookmark26)

[Concomitant Therapy 22](#_bookmark27)

[7.](#_bookmark28)

[7.1.](#_bookmark29)

[7.2.](#_bookmark30)

[7.3.](#_bookmark31)

[8.](#_bookmark32)

[8.1.](#_bookmark33)

[DISCONTINUATION AND WITHDRAWAL 22](#_bookmark28)

[Discontinuation of study intervention 22](#_bookmark29)

[Withdrawal from the study 22](#_bookmark30)

[Lost To Follow-Up 23](#_bookmark31)

[STUDY ASSESSMENTS AND PROCEDURES 23](#_bookmark32)

[Assessments 23](#_bookmark33)

[8.1.1.](#_bookmark34)

[Standard of Initial Asthma Care 23](#_bookmark34)

Version Date: June 25, 2025 (v3.0)

Page **3** of **54**


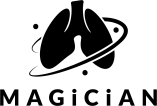


**MAGICIAN** Clinical Trial Protocol

[8.1.2.](#_bookmark35)

[8.1.3.](#_bookmark36)

[8.1.4.](#_bookmark37)

[Screening 23](#_bookmark35)

[Treatment Period 24](#_bookmark36)

[Follow-Up 24](#_bookmark37)

[9.](#_bookmark38)

[9.1.](#_bookmark39)

[9.2.](#_bookmark40)

[9.3.](#_bookmark41)

[9.4.](#_bookmark42)

[9.5.](#_bookmark43)

[9.6.](#_bookmark44)

[9.7.](#_bookmark45)

[9.8.](#_bookmark46)

[9.9.](#_bookmark47)

[9.10.](#_bookmark48)

[9.11.](#_bookmark49)

[9.12.](#_bookmark50)

[9.13.](#_bookmark51)

[9.14.](#_bookmark52)

[10.](#_bookmark53)

[10.1.](#_bookmark54)

[10.2.](#_bookmark55)

[10.3.](#_bookmark56)

[STATISTICAL CONSIDERATIONS 24](#_bookmark38)

[Sample size 24](#_bookmark39)

[The primary outcome measure 25](#_bookmark40)

[Secondary outcome measures 26](#_bookmark41)

[Other rare outcomes 26](#_bookmark42)

[Analyses: Primary efficacy analysis 26](#_bookmark43)

[Primary outcome 27](#_bookmark44)

[Secondary outcomes 27](#_bookmark45)

[Measurement of outcomes at follow up 27](#_bookmark46)

[Safety outcomes 27](#_bookmark47)

[Interim analysis 28](#_bookmark48)

[Subgroup analyses 28](#_bookmark49)

[Economic analysis 28](#_bookmark50)

[Adherence 28](#_bookmark51)

[Follow up loss 29](#_bookmark52)

[Adverse Events and Serious Adverse Events 29](#_bookmark53)

[Definition of Adverse Events (AE) 29](#_bookmark54)

[Definition of Serious Adverse Events (SAE) 29](#_bookmark55)

[Classification of an Adverse Event 30](#_bookmark56)

[10.3.1.](#_bookmark57)

[10.3.2.](#_bookmark58)

[10.3.3.](#_bookmark59)

[10.3.4.](#_bookmark60)

[10.3.5.](#_bookmark61)

[10.3.6.](#_bookmark62)

[11.](#_bookmark63)

[11.1.](#_bookmark64)

[11.2.](#_bookmark65)

[11.3.](#_bookmark66)

[11.4.](#_bookmark67)

[11.5.](#_bookmark68)

[11.6.](#_bookmark69)

[11.6.1.](#_bookmark70)

[Severity of Event 30](#_bookmark57)

[Relationship to Study Intervention 31](#_bookmark58)

[Expected adverse events 32](#_bookmark59)

[Time Period and Frequency for Event Assessment and Follow-Up 32](#_bookmark60)

[Reporting of Adverse Events 32](#_bookmark61)

[Serious Adverse Event Reporting 33](#_bookmark62)

[SUPPORTING DOCUMENTATION AND OPERATIONAL CONSIDERATIONS 34](#_bookmark63)

[Confidentiality and Privacy 34](#_bookmark64)

[Safety Oversight - Data Safety and Monitoring Committee (DSMC) 34](#_bookmark65)

[Participant Safety 35](#_bookmark66)

[Study Monitoring 35](#_bookmark67)

[Quality Assurance and Quality Control 36](#_bookmark68)

[Data Handling and Record Keeping 37](#_bookmark69)

[Data Collection and Management Responsibilities 37](#_bookmark70)

Version Date: June 25, 2025 (v3.0)

Page **4** of **54**


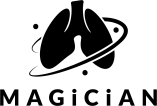


**MAGICIAN** Clinical Trial Protocol

[11.6.2.](#_bookmark71)

[11.7.](#_bookmark72)

[11.8.](#_bookmark73)

[Study Records Retention 37](#_bookmark71)

[Protocol Deviations 37](#_bookmark72)

[Publication and Dissemination of Results 38](#_bookmark73)

[APPENDIX A - Abstract from our MAGNUM RCT 39](#_bookmark74)

[APPENDIX B - Pediatric Respiratory Assessment Measure (PRAM)a score 41](#_bookmark75)

[APPENDIX C - Abstract from our secondary MAGNUM analysis 42](#_bookmark76)

[APPENDIX D - Abstract from our international PERN survey 44](#_bookmark77)

[APPENDIX E - Economic analysis 45](#_bookmark78)

[APPENDIX F - Annual Enrollment Projection 47](#_bookmark79)

[References 48](#_bookmark80)

Version Date: June 25, 2025 (v3.0)

Page **5** of **54**


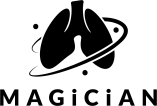


**MAGICIAN** Clinical Trial Protocol

**STATEMENT OF COMPLIANCE**

The trial will be conducted in accordance with this protocol, International Council on Harmonisation Good Clinical Practice (ICH GCP) and applicable regulatory requirements. The Principal Investigator (PI) will assure that both the Sponsor and the Research Ethics Board (REB) will be notified of protocol deviations in accordance with the Sponsor and local REB requirements.

The protocol, informed consent form(s), and all participant materials will be submitted to the REB for review and approval. Approval of both the protocol and the consent form(s) must be obtained before any participant is enrolled. Any amendment to the protocol will require review and approval by the REB before the changes are implemented to the study. All changes to the consent form will be REB approved; a determination will be made regarding whether a new consent needs to be obtained from participants who provided consent, using a previously approved consent form.

Name of Principal Investigator (Print):

Signature of Principal Investigator: Date:

<DD Month YYYY>

Site Address

Version Date: June 25, 2025 (v3.0)

Page **6** of **54**


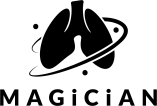


**MAGICIAN** Clinical Trial Protocol

**1.**

**PROTOCOL SUMMARY**

**1.1. Synopsis**

Version Date: June 25, 2025 (v3.0)

Page **7** of **54**

**Title:**

**Magnesium in acute pediatric asthma in the emergency department (MAGICIAN) - a multicentre randomized controlled clinical trial protocol**

**Study Description:**

This is a multi-centre, double blind, randomized controlled trial of intravenous magnesium in children with asthma or probable asthma. The purpose of this study is to find out if children who are given intravenous magnesium (IVMg) for refractory asthma/wheezing have a greater improvement in breathing discomfort compared to no magnesium. This protocol has not yet generated results.

We hypothesize that children between the ages of 2.00-17.99 with a Pediatric Respiratory Assessment Measure (PRAM) Score ≥ 5 that are given IVMg will have a mean PRAM improvement at 120 minutes post IVMg ≥1.0 point greater compared to placebo

**Objectives:**

Primary Objective:

- In children 2.00-17.99 years old who present to EDs with acute asthma and have persistent moderate-severe asthma after initial optimal standardized therapy, is there a greater reduction (improvement) in the PRAM score at 120 minutes post-intervention start in those given IVMg compared to placebo?

Secondary Objective:

Between these treatment modalities:

1. Is there a difference in the hospitalization rate for asthma at the index ED visit?
2. Is there a difference in the hourly changes in the PRAM, respiratory rate, heart rate oxygen saturation and blood pressure from the pre-intervention baseline to 180 minutes?
3. Is there a difference in the area under the receiver operating characteristic curve for PRAM within 120 minutes?
4. Is there a difference in the proportion of children achieving PRAM indicating mild asthma status, i.e. ≤ 3 points (widely accepted criterion for discharge home) at 120 minutes?
5. Does the treatment effect on the primary outcome vary between subgroups defined by: age, sex, pre-randomization PRAM, atopy, non-rhinovirus nasal pathogen36 and “viral- wheeze” phenotype?


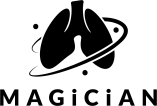


Enrollment closure: Winter 2027-2028, Analyses: Spring 2028

**MAGICIAN** Clinical Trial Protocol

Version Date: June 25, 2025 (v3.0)

Page **8** of **54**

1. Is there a difference in the hospitalization rate for asthma within 72 hours of ED discharge?
2. Is there a difference in the rate of unscheduled visits for asthma within 72 hours of ED discharge?
3. Is there a difference in the overall hospital length of hospital stay?

i) Is IVMg a cost-effective treatment option?

**Endpoints:**

Primary endpoint: PRAM score at 120 minutes post start of experimental therapy.

Secondary endpoints: PRAM, oxygen saturation, heart rate and resp rate 30, 60, 120, 180 minutes and blood pressure 10, 20, 30, 60, 120, 180 minutes after start of experimental therapy and unscheduled medical re-visits/hospitalizations for asthma 72 hours after ED discharge home.

**Study Population:**

192 children, ages 2.00-17.99 years, with a diagnosis of asthma or probable asthma presenting to the Emergency Department (ED) with a PRAM score ≥5 after receiving standard of care treatment for asthma.

**Phase:**

Phase III

**Description of Study Intervention:**

Two groups will be compared. One group will receive intravenous Magnesium and the other group will receive an intravenous placebo (salt water solution not containing magnesium) over 30 minutes.

**Study Duration:**

**Participation Duration:**

72 hours (~3 hours in the emergency department and then a brief email/telephone call ~72 hours later)


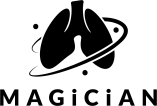

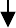

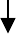

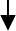

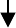

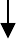

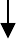


**MAGICIAN** Clinical Trial Protocol

**1.2. Schema**

**Triage Registered Nurse (RN):** Arrival PRAM score (**see Appendix B (PRAM Score)**)

Moderate to severe disease:

Assessment by ED physician Systemic corticosteroids

Initial therapy with 3 treatments with salbutamol & ipratropium (as per site drug dosing and ipratropium indications) over about 1 hour

Study nurse notified

**Study Nurse measures eligibility PRAM at end of initial therapy, if ≥ 5:** assesses eligibility (If eligibility PRAM is < 5, subject is ineligible for study)

If eligible, **Study Nurse** obtains informed consent

IV start, infusion of IV Normal Saline (NS) bolus as per site standard of care

**Study Nurse** retrieves next appropriate randomized treatment kit from research fridge/pharmacy department

**ED/Study Nurse** infuses experimental solution over 30 minutes

**Experimental Group Treatment**

**IV Mg sulfate** (**wt ≤33 kg: 75 mg/kg; >33 kg: 2,500 mg)**

**Control Group Treatment IV Normal Saline**

**Study Nurse** measures PRAM, RR, HR, BP, O2 sat @ Baseline (pre experimental Rx), BP@ 10’,20’,30’after start of experimental Rx), and PRAM, RR, HR, BP, O2 sat @ 30’,60’, 120’, 180’

Further bronchodilator/other therapy as clinically indicated after experimental infusion finished.

Disposition decision by ED MD

Electronic/paper health chart review and telephone/email follow-up at 72 hrs (+7days)

Version Date: June 25, 2025 (v3.0)

Page **9** of **54**


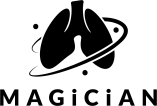


**MAGICIAN** Clinical Trial Protocol

**1.3. Schedule of Activities (SoA)**

The following schedule of activities are to be completed for each participant.

Footnote:

* Consent must take place before any study activities start.

† Only participants who are discharged from the ED are contacted to complete the survey.

Version Date: June 25, 2025 (v3.0)

Page **10** of **54**

Procedures

**Screening/Eligibility**

**Baseline (Pre-Intervention)** (Measurements completed as close to before giving intervention)

**Time Point 0** (administer intervention)

**Time Point 1 (Assessment 1)**

(10 minutes +/- 5 minutes)

**Time Point 2 (Assessment 2)**

(20 minutes +/- 5 minutes)

**Time Point 3 (Assessment 3)**

(30 minutes +/- 5 minutes)

**Time Point 4 (Assessment 4)**

(60 minutes +/- 15 minutes)

**Time Point 5 (Assessment 5)**

(120 minutes +/- 15 minutes)

**Time Point 6 (Assessment 6)**

(180 minutes +/- 15 minutes))

**Follow-Up** (72 hours after discharge - up to 7 days post ED discharge)

Informed Consent

X*

Demographics

X

Medical History

X

Randomization

X

Administer Study Intervention

X

Concomitant medication review

X

X

X

X

PRAM Score

X

X

X

X

X

X

Respiratory Rate

X

X

X

X

X

Heart Rate

X

X

X

X

X

Blood Pressure

X

X

X

X

X

X

X

O2 saturation

X

X

X

X

X

Survey

X†

Adverse Event review and evaluation

X

X

X

X

X

X

X

Complete Case Report Forms (CRFs)

X

X

X

X

X

X

X

X


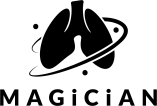


**MAGICIAN** Clinical Trial Protocol

**2.**

**INTRODUCTION**

**2.1.** **Study Rationale - The Need For A Trial**

Acute asthma exacerbations are a leading cause of pediatric emergency department (ED) visits and hospitalizations.1,2 Despite receiving optimal initial treatment with inhaled salbutamol, ipratropium and systemic corticosteroids (CS), 50% of the children with severe acute asthma experience ongoing moderate-to-severe respiratory distress.3 In this refractory population, which accounts for 84% of asthma hospitalizations, guidelines recommend intravenous magnesium (IVMg).4-9 However, IVMg therapy is resource- intensive as it demands close monitoring by specialized pediatric ED nurses, requires IV insertion, and can cause hypotension.10 To minimize these concerns, we evaluated the benefits of nebulized Magnesium (Mg) in a 7-centre Canadian CIHR-funded Randomized Controlled Trial (RCT) (MAGNUM) in this population.3 MAGNUM identified no benefits of inhaled Mg compared to placebo (**Appendix A**).3

There is highly limited and disparate evidence of benefit for IVMg in children, requiring further evidence to justify its continued use.11,12 Early evidence from 2 small single- center placebo-controlled pediatric RCTs (total N=61) suggested hospitalization benefit,13,14 while a third study reported no benefit (N=54).15 The most-recent Cochrane review based on these 3 studies expressed lack of confidence in the evidence prompted by a more appropriate re-analysis which yielded lack of IVMg effect on hospitalizations.16 Similar conclusion was reached by another review.17 Recent large observational studies found that IVMg was not associated with improved outcomes.11,18 A U.S-wide PHIS study of 91,000 children with acute asthma showed no association of IVMg with shorter hospital stay, lower initiation, duration of airway support or use of IV salbutamol.19 In a prospective study of pediatric moderate-severe refractory asthma with a propensity score-covariate adjustment, IVMg was associated with a 2-hour worsening

of the Acute Asthma Intensity Research Score, a 6-fold higher odds of hospitalization and no difference in time to every 4 hours salbutamol, an accepted parameter for discharge home, compared to no IVMg.11

These conflicting results may be due to differences in the timing of IVMg administration, confounding, and the choice and the timing of the primary outcome: 1) the early RCTs only measured an immediate IVMg effect on respiratory status;13,14,20-22 there is no evidence of its incremental benefit on outcomes measured at the peak effect of routine key co-interventions with CS and bronchodilators, when hospitalization is usually considered (approximately 4 hours after CS); 2) past RCTs did not employ validated asthma scores; 3) IVMg is usually given after ≥4 hours of unsuccessful ED treatment when most clinicians have already decided to admit, even if there is significant clinical improvement;19 4) children given IVMg have 6-10 times higher odds of hospitalization, in part due to confounding by indication, possible lack of Mg efficacy and physician discomfort with discharge after Mg due to concerns about rebound effects.11,23 In a secondary analysis of MAGNUM where some children required IVMg, we demonstrated that the decision to admit is independent of the presenting asthma severity and clinical response to IVMg,23 suggesting that some hospitalizations may be driven by physician

Version Date: June 25, 2025 (v3.0)

Page **11** of **54**


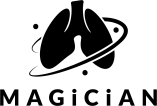


**MAGICIAN** Clinical Trial Protocol

discomfort about discharge after Mg rather than by true clinical Mg efficacy. Therefore, hospitalization does not represent an appropriate primary outcome to assess IVMg efficacy. In contrast, alleviation of respiratory distress is a highly suitable primary outcome, as it is the main reason for IVMg therapy.24

We propose an RCT [MAGnesIum Trial in ACute Asthma In Emergency DepArtmeNt: MAGICIAN trial] to conclusively determine if IVMg alters the asthma exacerbation course: it must be given early, with the improvement in respiratory distress measured at the peak effect of key co- interventions using a valid, discriminative, reproducible and responsive-to-change instrument, the Pediatric Respiratory Assessment Measure **(Appendix B: Pram Score)**.25

Trial results will impact care regardless of whether an association is found. If IVMg is found to decrease respiratory distress, we will use knowledge translation (KT) to implement *routine and early* IVMg therapy for refractory asthma. A negative result will be followed by de-implementation strategies to cease the use of IVMg for refractory asthma which may also lead to cost savings: IVMg therapy is personnel- intensive, requires an IV which may not be necessary in moderate-severe asthma, may cause hypotension,10 and as such greatly augments the odds of hospitalization.11,23

**2.2. Background**

Pediatric asthma guidelines recommend inhaled ß2 agonists, anticholinergics and systemic CS for acute attacks.4-6,8,9,26 However, 50% asthmatics are resistant to ß2 agonists3 and a response to CS may take well above 4 hours.27 In MAGNUM, 49% of screened children remained in moderate-severe distress after initial therapy and 45% were hospitalized.3 These non-responders represent the majority of asthma hospitalizations,25 with high treatment costs.28,29 IVMg sulfate could relieve airway obstruction by multiple mechanisms.30-33 Of the 8 RCTs evaluating IVMg in children, only 3 used a placebo-controlled design (N 31,30,54) assessing lung function/asthma score and hospitalization,13-15 with disparate conclusions. Two positive RCTs excluded preschoolers who constitute the majority of pediatric asthma ED visits34 and failed to use ipratropium for initial therapy,13,14 and the negative study did not limit enrolment to refractory asthma.15 The table below summarizes the pediatric RCTs of IVMg: 3 used non-placebo comparators, 1 did not measure hospitalization21 and 1 had no control group: only 3 small trials were placebo-controlled (shaded lines).

50 mg/kg/hr for 4 hours (max 8 gm IV Mg 50 mg/kg (no

Discharge home within 24 hours.

Clin asthma score at 1 hour Mechanical ventilation

Irazuzta

2015

38

6-18

in 4 hr)

max) over 1 hour IV terbutaline, IV aminophylline None

Singhi

*Torres*

2014

*2012*

100

*143*

1-12 50 mg/kg over 20 min (no max)

*2-15 25 mg/kg over 20 min (max 2 g)*

Version Date: June 25, 2025 (v3.0)

Page **12** of **54**

***Scarfone 2000 54 1-18 75 mg/kg over 20 min (max 2.5 g) Saline placebo*** Pulmonary Index at 2 hours

hospitalization

**Ciarallo 2000 30 6-18 40 mg/kg over 20 min (max 2 g) Saline placebo** PEFR, FEV1, FVC,

hospitalization

**Principal Year N Age IV-Mg Comparative Study Outcomes Author (years) therapy**


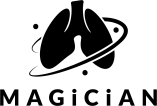


**MAGICIAN** Clinical Trial Protocol

Gurkan Devi

1999 20

1997 47

6-16 40 mg/kg over 20 min (max 2 g)

1-12 100 mg/kg over 35 min (no max)

Saline placebo Aminophylline

PEFR, vital signs at 1.5 hour PEFR and O2 saturation

PEFR, peak expiratory flow rate; FEV1, forced expiratory volume in 1 second; FVC, forced vital capacity

A review of 61,854 pediatric Emergency Department (ED) asthma visits from the U.S.PECARN registry found that only 26% of children hospitalized for asthma got IVMg in the ED,10 and that IVMg is generally given late (median 154 min).19 In our secondary MAGNUM analysis, IVMg was associated with a 10-fold increase in hospitalizations when adjusted for presenting and post Mg asthma severity,23 suggesting either confounding by indication, lack of Mg efficacy or concern about discharge after Mg due to potential rebound effect **(Appendix C)**. The short IVMg half-life (2-2.7 hrs)35,36 has not been linked to the duration of clinical effect of IVMg.17 Only 1.8% children discharged post IVMg return to the ED vs 3.6% without.10

Equipoise exists for IVMg benefit in refractory pediatric asthma.16 The latest 2016 Cochrane review highlights the limitations of the evidence, especially the small number of participants and disparate results.16 While the fixed-effect analysis of the 3 studies13-15 suggests a large reduction in hospitalizations, a re-analysis with a more appropriate random-effect method yielded lack of significant difference [OR 0.18 (0.02-1.59)] and while the point estimate is low, so was the authors’ confidence in the evidence.16

While IVMg itself is inexpensive, its administration is resource-intensive because of the need for nurses skilled in pediatric ED care and IVMg infusion protocols,37,38 as well as for blood pressure monitoring due to the potential for hypotension.39 Given challenges with IV access in some young children,40-44 ED physicians tend to delay IVMg therapy to avoid the “poke.”39 Clarification of IVMg benefit would provide a much-needed justification for related human and interventional resource use.

In preparation for this study, we conducted a Pediatric Emergency Research Canada (PERC) survey showing that a) alleviation of respiratory distress represents the main reason for IVMg use, b) most ED physicians routinely hospitalize after IVMg, irrespective of asthma severity or Mg response, in part due to lack of solid evidence of improved outcomes after IVMg **(Appendix D: PERN Survey)**.24

**2.3. Risk/Benefit Assessment - Participant Safety**

Mg has a theoretical potential for hypotension, hypopnea and heart block,45 but only hypotension has been reported with any frequency. The IVMg RCT of 1109 adults reported 8% hypotension46 but nobody had Mg stopped for hypotension. Pediatric IVMg trials with Mg doses 75 and 100 mg/kg did not report any hypotension (N=101).15,20 While a review of the PECARN Registry found a median dose 50 mg/kg had a hypotension rate of 6.8%,10 the mean decrease in the systolic pressure was 5 mm Hg, with uncertain clinical significance. No hypotension was observed in 3 pediatric pharmacologic ICU studies of 75 mg/kg IV Mg in children ≤30 kg and 50 mg/kg in those

>30 kg followed by 40 mg/kg/hour for 4 hours.47-49 Two of our participating sites

Version Date: June 25, 2025 (v3.0)

Page **13** of **54**

**Ciarallo 1996 31 6-18 25 mg/kg over 20 min (max 2 g) Saline placebo** PEFR, hospitalization


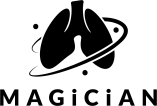


**MAGICIAN** Clinical Trial Protocol

routinely use 75 mg/kg IVMg. A literature review of pediatric status asthmaticus confirms lack of IVMg toxicity.50 Nonetheless, blood pressure will be measured at 10, 20, 30 and 60 minutes and hourly to 180 minutes. If the systolic pressure drops below the 5th percentile-for-age,51 necessary treatment such as IV fluids will be given. Because the children with unstable airway will be excluded and further inhaled salbutamol/other asthma therapies will be given if needed, lack of Mg in the placebo group will not endanger these participants. All study patients will be monitored for 180 minutes post- intervention prior to discharge, to ensure safety. Only 13/723(1.8%) children discharged post IVMg return to the ED vs 1383/38,623(3.6%) without; IVMg is not associated with re-visits.10

**3.**

**OBJECTIVES AND END POINTS**

**Primary question:** In children 2.00-17.99 years old who present to 6 PERC EDs with acute asthma and have persistent moderate-severe asthma after initial optimal standardized therapy, is there a greater reduction (improvement) in the PRAM score at 120 minutes post- intervention start in those given IVMg (75 mg/kg, max 2.5 g), compared to placebo?

**Primary endpoint:** PRAM score at 120 minutes post start of experimental therapy.

**Secondary questions:** Between these treatment modalities:

a)

b)

Is there a difference in the hospitalization rate for asthma at the index ED visit?

Is there a difference in the hourly changes in the PRAM, respiratory rate, heart rate oxygen saturation and blood pressure from the pre-intervention baseline to 180 minutes?

Is there a difference in the area under the receiver operating characteristic curve for PRAM within 120 minutes?

Is there a difference in the proportion of children achieving PRAM indicating mild asthma status, i.e. ≤ 3 points (widely accepted criterion for discharge home) at 120 minutes?

Does the treatment effect on the primary outcome vary between subgroups defined by: age, sex, pre-randomization PRAM, atopy, non-rhinovirus nasal pathogen36 and “viral-wheeze” phenotype?

Is there a difference in the hospitalization rate for asthma within 72 hours of ED discharge?

Is there a difference in the rate of unscheduled visits for asthma within 72 hours of ED discharge?

Is there a difference in the overall hospital length of hospital stay? Is IVMg a cost-effective treatment option?

c)

d)

e)

f)

g)

h)

i)

**Secondary endpoints:** PRAM, oxygen saturation, heart rate and resp rate 30, 60, 120, 180 minutes and blood pressure 10, 20, 30, 60, 120, 180 minutes after start of experimental therapy and unscheduled medical re-visits/hospitalizations for asthma 72 hours after ED discharge home.

Version Date: June 25, 2025 (v3.0)

Page **14** of **54**


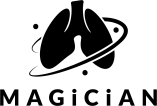


**MAGICIAN** Clinical Trial Protocol

**Hypothesis:** Based on a two-tailed hypothesis, children with PRAM ≥ 5 after initial therapy given IV Mg will have a mean PRAM improvement at 120 minutes post IVMg ≥1.0 point greater compared to placebo.

**4.**

**STUDY DESIGN**

**4.1. Overall Design and the Scientific Rationale for Study Design**

As mentioned above, previous RCTs of IV Mg in children are small and have methodologic limitations. The most recent systematic review (Griffiths, 2016) concludes there is equipoise about IV Mg benefit. More recent large IVMg studies cast doubt on Mg benefit but these have non-RCT designs. Therefore, we propose a 6-centre randomized, double-blind, placebo-controlled trial. Two groups will be compared: IVMg sulfate and IV0.9% saline placebo. After initial therapy with the systemic CSs routinely used for acute asthma management at a given site, 3 treatments with inhaled salbutamol and ipratropium (indications for ipratropium vary by site),52 eligible patients with PRAM ≥5 **(Appendix B: PRAM Score)** will receive a 30-minute IV infusion of 75 mg/kg of Mg sulfate (maximum 2.5 g) [experimental group] or an identical volume of 0.9% saline [control group]. Outcomes will be measured during the 180-minute observational period in the ED and at 72 hours post ED discharge. Primary outcome will be the PRAM score at 120 minutes after start of experimental therapy.

PRAM: PRAM is a validated 12-point asthma severity score53 which exhibits the most comprehensive measurement properties of all asthma scores54 and has been successfully used as an outcome in major trials.3,55 It is the only score with demonstrated criterion validity, using respiratory resistance as the gold standard.25,53 PRAM has been validated in both preschool and school-aged children in the ED with asthma and has strong association with admission.25 PRAM has inter-rater reliability above 70%25 and is adopted in all pediatric EDs in Canada. Most children treated for acute asthma are preschoolers56 who lack coordination to perform pulmonary function tests reliably. To maximize the accuracy of the PRAM measurement, all study nurses will complete online PRAM training module57 at https://enseignement.chusj.org/PRAM-En. We will use an eligibility cut-off of PRAM ≥ 5 post initial therapy as this is associated with clinically concerning respiratory distress requiring further intervention. PRAM ≥ 5 after one hour of initial care has high accuracy (AUC: 0.88) to predict hospitalization.58 Of the 215 children given IVMg in MAGNUM, 118 had PRAM 5, 41 PRAM 6, 35 PRAM 7 and 21 PRAM ≥8 prior to IV Mg.3

**4.2. End of Study Definition**

A participant is considered to have completed the study if he or she has completed all phases of the study including the last scheduled procedure shown in **Section 1.3(SoA)**.

A participant who is discharged home at the index ED visit will have relevant outcome measures completed by the study nurse up to 180 minutes. These participants will also complete a follow-up questionnaire at approximately 72 hours, at which point their study will be complete.

Version Date: June 25, 2025 (v3.0)

Page **15** of **54**


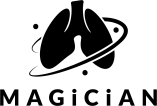


**MAGICIAN** Clinical Trial Protocol

A participant who is admitted to the hospital (e.g transferred from ED to an in-patient unit) will have the study nurse complete any study measures up to 180 minutes. After that, the participant is considered complete. No follow-up will occur 72 hours later.

**5.**

**STUDY POPULATION**

**5.1. Inclusion criteria:**

1. Age 2.00-17.99 years (prior to 18th birthday),
2. Diagnosis of asthma, defined as an asthma or probable asthma diagnosis/asthma- like phenotype made by a physician (this includes ED physician) in a patient who in the opinion of the treating ED physician requires therapy for acute asthma in the ED (GINA asthma guidelines, 2024).59
3. Moderate-severe asthma after initial therapy with 3 treatments of inhaled salbutamol and ipratropium, defined as an eligibility PRAM ≥5, indicating a strong association with hospitalization.25

**5.2. Exclusion criteria:**

1)

*2)*

Receipt of IVMg within 24 hours prior to ED arrival.

Need for airway support on arrival. *(Airway support on arrival meeting exclusion criteria will include immediate need for high flow nasal cannula therapy, non- invasive CPAP/bi-PAP ventilation or invasive ventilation with endotracheal intubation, as decided by the attending Emergency Department (ED) physician. Supplemental oxygen therapy will not represent an exclusion criterion.)*

Known renal, chronic pulmonary, neurologic, cardiac or systemic disease: *these may influence outcomes after Mg*.

Known hypersensitivity to Mg sulfate. Previous enrollment in this study.

Poor mastery of English and/or French language precluding informed consent understanding.

No phone/email; unavailable for follow-up

3)

4)

5)

6)

7)

**5.3. Strategies For Recruitment and Retention**

We expect 6 Canadian Hospitals to participate in this study. The anticipated study duration is approximately 38 months. We anticipate an 8-month pre-enrollment period, patient recruitment will occur for approximately 26 months, and an estimated 4-month post-enrollment period.

The target sample size is 192 participants. We anticipate an accrual rate of 7-8 participants a month in Emergency Departments (ED) across all sites.

Children presenting to the collaborating EDs who meet eligibility criteria will be approached for enrollment when the research nurses are on duty/on call (days and

Version Date: June 25, 2025 (v3.0)

Page **16** of **54**


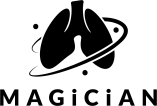


**MAGICIAN** Clinical Trial Protocol

evenings). Participating sites have research assistants in the ED who will pre-screen potentially eligible patients and ensure the miss rate is kept to a minimum.

As per our previous MAGNUM trial with a similar design, we estimate that only 15% of screened patients will participate: 75% will be ineligible and 50% of the remaining will decline consent or not join for other reasons.3 We shall adopt the MAGNUM enrollment schedule, with coverage by on-call research nurses, to enroll 192 patients with full data across 6 sites in approximately 26 months. This is achievable given that MAGNUM used the same eligibility criteria and entry asthma severity and randomized 729 children with full data at 7 centers in 5 years (21 patients/year/site). Thus, we anticipate annual enrollment of 109 eligible consenting children with full data based on annual asthma ED presentations **(Appendix F)** and a conservative estimate of 30% weekly hours coverage, 25% eligibility, 50% of those eligible not participating for refusals/other reasons, 5% non-compliance with intervention (crossover) and 1% loss to follow up.

**6.**

**STUDY INTERVENTION**

**6.1. Study Intervention Description**

Previously healthy children 2.00-17.99 years of age with acute asthma will have PRAM routinely measured in triage, as per usual practice. Children presenting with a PRAM ≥ 5 will be considered potentially eligible and will receive oral dexamethasone, predniso(lo)ne or IV CS in triage/shortly thereafter. They will also receive three salbutamol and ipratropium inhalations61 via Metered Dose Inhaler/valved holding chamber approximately 20 minutes apart, with medication doses and ipratropium indications depending on local practice. If the PRAM is ≥ 5 after the first bronchodilator treatment and the child has no co-morbidities,62 a consent to approach for research will be obtained. At the conclusion of the 3 baseline inhalations, the research nurse will confirm eligibility, confirm the eligibility PRAM score, and obtain informed consent. A topical anesthetic may be used at the IV if that is the practice at a given site and IV line will be inserted. Participants may also receive an IV fluid bolus, as per site ED practice, prior to the experimental therapy, to improve hydration and prevent hypotension.

Thereafter, the trained and intervention-blinded study nurse will enter the patient eligibility data into the database, obtain the next study kit assigned to the appropriate age group (<6th birthday vs age 6 years and older) from the study fridge and enter the study kit number in the database. Master lists will be provided to all participating research pharmacies which will permit the preparation of the study kits according to the randomization schedule. Prior to the start of the experimental therapy, the study nurse will measure the patient’s Baseline PRAM, respiratory rate, heart rate, blood pressure and oxygen saturation.

**6.2. Study procedures (Dosing and Administration)**

The study nurse will administer the assigned experimental therapy (IV Mg sulfate

Version Date: June 25, 2025 (v3.0)

Page **17** of **54**


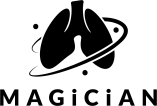


**MAGICIAN** Clinical Trial Protocol

or normal saline placebo) over a 30-minute period. The patients, study nurses, ED physicians and investigators will be blinded to treatment assignment; the pharmacy will be unblinded. [The SickKids research pharmacist will provide a manual with instructions on how each pharmacy will prepare blinded numbered kits with Mg or 0.9% saline placebo, according to routine IVMg sulfate infusion protocols (**Section**

**6.4.3 Logistics of Blinding and Kit Making)**. Each site will receive requirements for drug accountability and handling].

**Mg dose:** The chosen Mg sulfate dose of 75 mg/kg is at the upper end of the recommended dose range63,64 and is needed to minimize the probability that a subtherapeutic dose led to lack of observed treatment effect. Children weighing ≤30 kg require larger IVMg dose/kg than their heavier counterparts.47,48 Therefore, we shall administer IVMg 75 mg/kg (up to 2.5 g) to patients weighing ≤33 kg and 2.5 g to those >33 kg. This regime has a reassuring safety record: two pediatric studies of IVMg have employed doses of 75-100mg/kg without adverse effects.15,20 In three pediatric studies of 75 mg/kg IVMg bolus followed by 40 mg/kg/hour for 4 hours, no child developed hypotension.47,48 Two participating sites (Ste Justine’s, McMaster) routinely use 75 mg/kg IVMg.

**6.3. Study Procedures (after Administration of Study Intervention)**

Following the 30-minute experimental infusion, participants will continue to receive inhaled salbutamol, supplemental oxygen and other asthma medications as clinically warranted. *To prevent contamination, the use of open-label IVMg will be strongly discouraged* until after primary outcome determination at 120 minutes. In the highly exceptional event of respiratory failure after the intervention, immediate open-label IVMg will be permitted with locally used Mg doses (**Section 6.6 Emergency Unblinding Procedure)**, but unblinding will not be needed because a 2nd Mg infusion is safe for normotensive patients.47-49,51,60 Because children with unstable airway will be excluded and other routine asthma co-therapies will be given if needed, we do not anticipate open-label IVMg use prior to this time. IV in the placebo group is both clinically reasonable and ethically defensible: as the eligible children have refractory asthma, their ongoing respiratory distress will justify an IV to provide the often inadequate hydration and a potentially beneficial therapy to alleviate respiratory distress. Many of these children will need to be hospitalized which is often accompanied by IVMg therapy, other IV bronchodilators, and hydration. Topical anesthesia may be used if that is the practice at a given site.

Disposition will be determined by intervention-blinded ED physicians. While there is no known association between Mg serum levels and the duration of Mg bronchodilator effect, all study patients will be observed for 3 hours, to ensure safety. As per guidelines, discharged patients will be prescribed inhaled salbutamol, oral CS and inhaled CS, using locally employed regimes and doses. Further care will be sought if salbutamol is given more often than every 4 hours or if respiratory status interferes with the usual activity. There will also be a standardized electronic/telephone follow up to ascertain outcomes at 72 hours post ED discharge

Version Date: June 25, 2025 (v3.0)

Page **18** of **54**


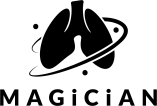


**MAGICIAN** Clinical Trial Protocol

home.

**6.4. Preparation/Handling/Storage/Accountability**

**6.4.1.**

**Acquisition and Accountability**

Pharmacies at each of the participating sites will be using IV Magnesium Sulfate and Normal Saline that is routinely used through their hospital supply and available in the Canadian market to prepare the study kits to be used for blinded randomization.

**6.4.2.**

**Preparation of Treatment allocation and storage**

The Hospital for Sick Children research pharmacy department will produce Master Randomization tables through randomize.net, stratified by site and age group (< 6years old vs ≥6 years old),52 employing permuted randomization of varying block sizes in a 1:1 ratio of Mg sulfate to placebo. Upon receiving consent, the study nurse will enter the patient eligibility data into the database and will obtain the next assigned study kit.

Master lists will be provided to all participating research pharmacies which will permit the preparation of the study kits according to the randomization schedule. Because IV magnesium sulfate has to be refrigerated at 2-8˚C, the experimental kits containing either IV Mg or normal saline will be stored in the ED research fridge.

**6.4.3.**

**Logistics of Blinding and Kit Making**

Each site will prepare consecutively numbered randomization kits, numbered

according to the site’s Master Randomization table.

Each kit will contain:

Version Date: June 25, 2025 (v3.0)

Page **19** of **54**

**Arm**

**Investigational Drug or Placebo**

**(Blinded Vial in Kit)**

**Dose**

**Active**

Magnesium Sulfate Injection

500mg/mL vial (5.5mL)

75 mg/kg, max 2.5 grams (x ml = y mg)

**Placebo**

Normal Saline (0.9%) injection vial (5.5mL)

Weight-based volume to be identical to Mg solution

(x ml = y mg)


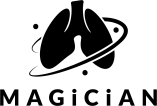


**MAGICIAN** Clinical Trial Protocol

•

Magnesium Sulfate Injection 500mg/mL OR 0.9% sodium chloride injection

•

**Active Kits** will contain Magnesium Sulfate injection

•

•

Injection to be administered by intravenous administration Unblinded site pharmacy will repackage small batches of Canadian commercial magnesium sulfate injection (5.5mL) into 10mL empty sterile vials in a laminar air flow hood according to a detailed worksheet procedure in the Pharmacy Manual of Operations.

•

**Placebo Kits** will contain 0.9% sodium chloride injection

•

Unblinded site pharmacy will prepare small batches of Normal Saline (0.9%) injection (5.5mL) into 10mL empty sterile vials in a Laminar Air Flow hood according to a detailed worksheet procedure in the Pharmacy Manual of Operations.

•

The repackaged Magnesium Sulfate and compounded placebo vials will be given a 3-month expiry date.

During Kit assembly by the site pharmacy, identical labels will be placed on the blinded vials to ensure the integrity of the blind.

Blinded Numbered Randomization Kits will be assembled by the unblinded site pharmacy and made available to the Emerg Study RNs for storage in the research fridge/pharmacy department for use once a subject is eligible to be randomized.

•

•

In this Investigator initiated study, the numbered kits will be assembled and labeled in the local Research Pharmacy according to detailed kit making Standard Operating Procedures provided by the Coordinating Pharmacy at SickKids. All kits/products will have appropriate Clinical Trial labeling according to Canadian regulations.

**6.5. Measures to Minimize Bias: Randomization and Blinding**

**Treatment allocation:** The leading site pharmacy will use the randomize.net tool to produce the study Master Randomization tables, stratified by site and age group (< 6years old, i.e. prior to 6th birthday vs ≥6 years old as pre-schoolers may respond differently to asthma therapies),52 employing permuted randomization of varying block sizes in a 1:1 ratio of Mg sulfate to placebo. The study nurse will transcribe the assigned study kit numbers into the secure Health Canada-validated REDCap database.

Master lists will be provided to all participating research pharmacies which will permit the preparation of the study kits according to the randomization schedule.

Version Date: June 25, 2025 (v3.0)

Page **20** of **54**


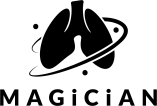


**MAGICIAN** Clinical Trial Protocol

The patients, outcome assessors (study nurses, ED physicians) and investigators will be blinded to treatment assignment; the pharmacy will be unblinded. The SickKids research pharmacist will provide a manual with instructions on how each pharmacy will prepare blinded numbered kits with Mg or 0.9% saline placebo, according to routine IVMg sulfate infusion protocols (**Section 6.4.3 Logistics of Blinding and Kit Making)**. Each site will receive requirements for drug accountability and handling. Unblinding is unlikely and did not occur in MAGNUM. The active Mg and saline placebo are identical in volume, color, odor, and appearance. Further, children with unstable airway will be excluded and only 6% experience a mean BP drop 5mmHg after IVMg with fewer requiring intervention.10

Administration of open-label IV-Mg would highly increase the probability of study contamination and will be strongly discouraged until after 120 minutes after the start of the experimental therapy. Children not responding to/worsening after the experimental therapy will be given back-to-back administration of inhaled salbutamol, other required medications and supplemental oxygen if necessary. In the highly exceptional event of impending respiratory failure after the intervention (children with unstable airway on arrival will be excluded), immediate open-label IVMg will be permitted with locally used Mg doses, but unblinding will not be needed because a 2nd Mg infusion is safe for normotensive patients.47-49,51,60 (**Section**

**6.6 Emergency Unblinding).**

**6.6. Emergency Unblinding Procedures**

Unblinding should only be requested when the clinical treatment of the patient will be different by knowing which arm of the study the patient was previously on. This may occur in a highly exceptional scenario of a suspected respiratory failure accompanied by clinically significant hypotension requiring treatment during the experimental therapy (presumed to have occurred during experimental Mg), and continuation of the experimental therapy would be undesirable. Hypotension will be defined as systolic blood pressure <70 mm Hg +(age in years x2) in children 2-10 years old and as <90 mm Hg in those

>10 years old. The study PI/local PI and the study nurses will remain blinded.

Hypotension occurring after the experimental therapy will be treated as needed but does not require unblinding.

The following Emergency Unblinding procedure will be followed:

1. Treating Physician or RN should contact the local QI of the study for consultation to unblind. In the event they cannot be reached immediately go to the next step.
2. Contact the SickKids hospital pharmacy by phone. The Research Pharmacy is available Mon – Fri from 08:00 to 16:00 EST at 416-813-6705 ext. 1 and ask to speak to a Research Pharmacist. At all other times or on statutory holidays call 416-813-6699 and ask to speak to a Pharmacist about an unblinding.
3. Provide the patient’s study randomization number, reason for unblinding, your site and your name to the SickKids pharmacist who will then provide the unblinded study arm.

Version Date: June 25, 2025 (v3.0)

Page **21** of **54**


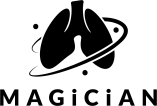


**MAGICIAN** Clinical Trial Protocol

1. Note that all patients whose therapy is unblinded must stop receiving the experimental therapy The ED physician will prescribe additional treatment as clinically appropriate.
2. The requesting physician should initiate Email communication within 24 hours detailing the request for Emergency unblinding and why. The email must inform the local PI and SickKids Research Pharmacist and Study Principal Investigator ([suzanne.schuh@sickkids.ca](mailto:suzanne.schuh@sickkids.ca))
3. The DSMC and REB will be advised of emergency unblinding within 48 hours of Sponsor becoming aware of the incident.

**6.7. Concomitant Therapy**

We are collecting medications given prior to arrival in the ED. This is to compare groups at baseline. During the study intervention of 30 minutes no other medications will be given. Following the 30-minute experimental infusion, participants will continue to receive inhaled salbutamol, supplemental oxygen and other asthma medications as clinically warranted. These medications will be collected as outlined in section 1.3 (SoA) in the case report form.

**7.**

**DISCONTINUATION AND WITHDRAWAL**

**7.1. Discontinuation of study intervention**

In the exceptional event the patient develops hypotension requiring therapy, apnea, or another adverse event reasonably attributable to Mg and the ED physician feels that the experimental therapy cannot be safely continued, the experimental treatment will be stopped and appropriate treatment given. Hypotension will be defined as systolic blood pressure <70 mm Hg +(age in years x2) in children 2-10 years old and as <90 mm Hg in those

>10 years old.

Lack of clinical improvement/deterioration after experimental therapy will be treated with administration of supplemental oxygen (if needed) and further inhaled salbutamol ± ipratropium treatments. Hypotension after experimental therapy will be treated with supplemental IV fluids/other measures, as per usual clinical practice. Neither of these scenarios apply to discontinuation of study intervention.

**7.2. Withdrawal from the study**

Participants are free to withdraw from participation of this study at any time upon request. An Investigator may discontinue or withdraw a participant from this study for the following reasons:

•

Withdrawal of informed consent (participant or parent/guardian withdraw for any reason)

•

If the participant’s blood pressure drops below the 5th percentile during the experimental infusion (this needs to be confirmed with manual BP measurement) and the treating physician feels that treatment is required,

Version Date: June 25, 2025 (v3.0)

Page **22** of **54**


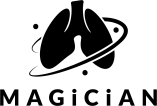


**MAGICIAN** Clinical Trial Protocol

experimental infusion will be stopped but the child will remain in the study and study allocation will remain blinded. Participants who develop hypotension or receive open-label IVMg during the 180-minute observational period will also remain in the study without unblinding. Participants whose treatment assignment has been unblinded will also remain in the study.

**7.3. Lost To Follow-Up**

We anticipate virtually no loss to follow-up for the primary outcome because the primary endpoint will happen in the ED. Participants discharged home from the ED will be contacted via email or phone (as per the preference of the family) 72 hours after discharged for a follow-up survey. The site will make every effort to contact the participant daily up to 7-days after discharge.

If the participant is unable to be reached via phone or email (their preferred method of contact) within 7 days after discharge, we will consider them lost to follow-up for this secondary outcome. They will not be withdrawn from the study, we will record in the study database that we were unable to contact participant for the final study time point.

**8.**

**STUDY ASSESSMENTS AND PROCEDURES**

**8.1. Assessments**

The schedule of activities is provided in section 1.3 (SoA).

All relevant data will be captured on paper or electronic case report forms (eCRFs) in accordance with the eCRF completion guidelines.

**8.1.1.**

**Standard of Initial Asthma Care**

When a potential participant arrives in the ED with asthma/wheezing, the Standard of Care (SOC) for this population is for the triage nurse to take an arrival PRAM score, followed by administration of systemic corticosteroids, and 3 treatments with inhaled salbutamol and ipratropium over the course of approximately 1 hour.

**8.1.2.**

**Screening**

The study team will pre-screen all potential participants who arrive at the ED. The study nurse may be contacted if a participant is identified with a PRAM ≥ 5 after the first inhalation, if the child had no past co-morbidities, and had at least one past wheezing episode.

After approximately 1 hour of SOC, the study nurse will approach participant to discuss the study and measure an eligibility PRAM score. If the participant is

Version Date: June 25, 2025 (v3.0)

Page **23** of **54**


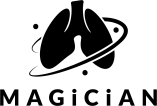


**MAGICIAN** Clinical Trial Protocol

eligible, they will screen all inclusion/exclusion, obtain informed consent, obtain history and demographics information.

**8.1.3.**

**Treatment Period**

The ED staff or study nurse, may use a topical anesthetic to start an IV if that is the practice at a given site. Prior to experimental therapy, they may also infuse IV Normal Saline bolus, according to site routine practices. The experimental infusion will last approximately 30 minutes. The study nurse will perform activities outlined in Section 1.3 (SoA) during this period.

**8.1.4.**

**Follow-Up**

Participants will receive a follow-up phone call or email (they may state their preference) 72 hours after they are discharged from the ED from the study team.

The study team may attempt to contact participants up to 7 days after discharge to answer the follow-up survey.

If the participant was admitted to the hospital at their index ED visit, they will not be contacted for the follow-up survey.

**9.**

**STATISTICAL CONSIDERATIONS**

**9.1. Sample size**

Sample size is based on the assessment of the PRAM change in the IVMg vs the control group and we *conservatively* aim for an inter-group PRAM difference in change of ≥1.0 point (SD 2.5), based on the following evidence. Children 1-17 years old with a baseline PRAM ≥4 points had a median PRAM change of 3 points if discharged vs.1 point if admitted.25 In a PRAM validation study of children with moderate dyspnea anchored against expert physician-perceived clinical improvement a PRAM change of 1.5 (SD 2.3) was seen for children judged clinically improved vs. 0.0 (SD 2.2) for those unchanged.67 When compared to a change in respiratory rate percentile, a PRAM change of 1.1 (SD 2.7) was seen in the improved patients vs.0.3 (SD 2.2) in those unimproved.67 Based on this evidence, the investigators agree that a minimally clinically significant difference in PRAM changes between groups is 1 point. Although the study non- compliance and loss to follow-up rates in MAGNUM were both 0%, we conservatively assume that the non-compliance (crossover) with allocated therapy and the follow up loss may be as high as 5% and 1%, respectively. Based on these parameters, a study sample of 192 children (96 per group) provides 80% power with a two-sided significance level 0.05. Accounting for the randomization factors of site and age, a sample of 192 children obtained by recruiting in 12 strata (2 ages x 6 sites) with a similar number of children per strata, a significance level of 0.05 and an intracluster correlation coefficient of 0.05, achieves 80% power to detect PRAM difference in change of 1.0 (SD 2.3) based on a mixed-model analysis.

Version Date: June 25, 2025 (v3.0)

Page **24** of **54**


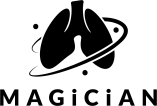


**MAGICIAN** Clinical Trial Protocol

**9.2. The primary outcome measure**

The primary outcome measure will be the PRAM score,25,53 measured at 30, 60, 120 and 180 minutes post intervention, with the 120 minute measurement as the primary end- point. PRAM is the best tool to ascertain clinical response in this trial. It is routinely used in pediatric EDs across Canada and abroad to guide asthma therapy and will: a) help identify eligible candidates, b) measure the incremental benefit of IVMg across sites, c) promote acceptance of trial results by the end-users, d) provide greater sensitivity to change than spirometry, and e) facilitate implementation of findings in Canada and beyond.

Two ED studies (N=200) of children with moderate-severe acute asthma confirmed that the PRAM score at 3 hours post initial management accurately predicted hospitalization/long ED stay (AUC 0.85 to 0.88, OR 2.0) as well as clinical improvement (our primary end-point).58,65 This time point also corresponds to 4 hours post initial CS therapy when CS is expected to act and hospitalization decisions are usually made. PRAM is a 12-point instrument validated in children 1-17 years old with acute asthma in the ED exhibiting the most comprehensive measurement properties of all pediatric acute asthma scores,54 which was successfully used as an outcome in major trials.3,55 PRAM is the only pediatric acute asthma score developed for pre-schoolers by criterion (oscillometry) and construct (physician severity appraisal) validity.25,53 PRAM also demonstrates good discrimination and responsiveness to change: a construct- validation (hospitalization) in 800 children aged 1-17 years (oscillometry and physician appraisal) showed good internal consistency (Cronbach α 0.7), responsiveness (Guyatt responsiveness index 0.7), and inter-rater reliability across ages.25,53 PRAM is one of two scores that performed best in validation, responsiveness, and reliability in the two systematic reviews.54,66 Two prospective validation studies compared the psychometric properties of the highest-rated clinical scores in 50 children.67,68 One found PRAM as one of two most valid (construct and content-wise) scores of 36 dyspnea scores. The inter-rater reliability was modest, because of no rating of the auscultation and palpation criteria, which represented 3 of 5 items.67 The second study rated PRAM as second-best for predicting hospitalization (AUC 0.89).68 Because the raters were not PRAM-trained, the inter-rater reliability was lower than in the primary studies (weighted kappa 0.50): this will be remedied by a mandatory participation in an online training module57 by all research nurses. While PRAM detected significant and meaningful changes in ED treatment from baseline to 4 hours in school-aged children, spirometry did not, suggesting a higher PRAM sensitivity to change.69 Unlike spirometry, PRAM will allow inclusion of preschoolers who represent 60% of asthma visits to pediatric EDs and 40% of the school-aged children unable to perform spirometry.56

*We considered but rejected using hospitalization as the primary outcome as it would likely artificially attenuate inter-group difference and mask a true difference in clinical improvement.* Hospitalization is a subjective decision influenced by asthma severity, hospital policy and provider risk-tolerance. Further, major confounding by indication is

Version Date: June 25, 2025 (v3.0)

Page **25** of **54**


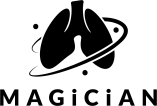


**MAGICIAN** Clinical Trial Protocol

likely.11,23 Finally, a meaningful PRAM effect size would provide a compelling reason for *routine early use* of IVMg, regardless of hospitalization, while hospitalization is irrelevant with a null or negative result.

The 120-minute time point includes the initial asthma therapy (one hour), subsequent period involving eligibility confirmation, informed consent, IV placement and pre- hydration (approximately one hour) and the two-hour period after the start of experimental therapy. Because corticosteroids are typically given at the start of the initial therapy, the 120-minute point will occur approximately 4 hours post corticosteroids when most of the improvement after corticosteroids occurs (Alnaji et al, Acad Emerg Med 2014).

**9.3. Secondary outcome measures**

The two study groups will also be compared with respect to:

1. Hospitalization for asthma at the index ED visit.
2. Changes in PRAM, respiratory rate, heart rate, oxygen saturation and blood pressure from baseline (pre- intervention) to 30, 60, 120, and 180 min and also in blood pressure to 10 and 20 min post-intervention
3. Area under the receiver operating characteristic curve for the PRAM score changes at 30, 60, and 120 minutes.
4. PRAM denoting mild asthma (≤ 3 points is a widely accepted discharge criterion25) at 120 minutes
5. Hospitalization for asthma at any medical facility within 72 hours post- ED discharge.
6. Unscheduled asthma-related visits to any health care provider within 72 hours post- ED discharge.
7. Hospital length of stay.
8. Open label IVMg administration after the 120-minute measurement of the primary outcome.

**9.4. Other rare outcomes**

While the study cannot be powered for their meaningful analysis, we will collect

a) ICU admissions, b) safety outcome of actionable hypotension (systolic blood pressure <5th percentile for age51 treated with IV fluids/other therapy) **(Section 10 Adverse and Serious Adverse Events)**. While many physicians claim to use IVMg to decrease ICU admissions, only 5% (19/372) of hospitalized children received ICU care in MAGNUM.3

**9.5. Analyses: Primary efficacy analysis**

We will adopt an intention-to-treat (ITT) approach and a sensitivity analysis using a per- protocol (PP) approach. Variables will be reported using frequencies and proportions; and continuous variables will be expressed as means and standard deviations or medians and interquartile ranges in case of non-normal distribution. Baseline variables will be compared between the IVMg vs placebo using standardized differences (SD) with an absolute SD < 0.10 being considered balanced.

Version Date: June 25, 2025 (v3.0)

Page **26** of **54**


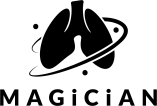


**MAGICIAN** Clinical Trial Protocol

**9.6. Primary outcome**

For the primary outcome of the change in PRAM at 120 minutes, the study groups will be compared using the Student’s t-test. A comparison based on a multiple regression analysis will be conducted adjusting for the randomization stratification factors site and age.

**9.7. Secondary outcomes**

For the continuous outcomes changes in the PRAM, respiratory rate, heart rate and oxygen saturation from pre-intervention to 30, 60, 120, and 180 min, a mixed model repeated measures will be conducted with treatment group as the between factor (IVMg vs placebo) and the within factor time 0,60,120,180 min. The analysis will be repeated with the randomization stratification factors site and age. Similarly, blood pressure will be analyzed with the additional time measures at 10, 20 and 30 minutes. For overall hospital stay, the study groups will be compared using the Wilcoxon ranked-sum test. For binary outcomes (hospitalization, PRAM indicating mild asthma, unscheduled asthma-related re-visits), groups will be compared using the chi-square test. For the analysis of the area under the ROC curve for PRAM scores, a mixed model repeated measures ANOVA will be conducted with the between factor study group (intervention vs placebo) and within factor time (30, 60, 120 minutes). In addition to the omnibus test for the group and time factors, an orthogonal polynomial analysis will be conducted to compare trends over time between the study groups.

A comparison based on a logistic regression analysis will be conducted adjusting for the randomization stratification factors site and age. As the secondary analyses are exploratory, overall significance for them will be set at 0.05 (2-sided).

**9.8. Measurement of outcomes at follow up**

Trained and intervention-blinded research nurses will measure PRAM, respiratory rate, oxygen saturation and blood pressure pre-randomization and at the above intervals, up to 180 minutes post starting the intervention, ascertain disposition and other pharmacotherapy given in the ED and determine other outcomes via automatic REDCap e- mails/study team e-mail/telephone at 72 hours post-ED discharge. If there has been no response, there will be daily reminders until day 7.

**9.9. Safety outcomes**

Each adverse event will be described as per good clinical practice guidelines. Descriptive statistics will be used to report frequencies of adverse events and SAEs in the groups. If appropriate, differences between the groups will be compared using a two-sample t-test or Wilcoxon rank sum test. These analyses will be reviewed by Data and Safety Monitoring Committee (DSMC) every 6 months.

***Missing Data:*** Missing data will be imputed using multiple imputation. Analysis

Version Date: June 25, 2025 (v3.0)

Page **27** of **54**


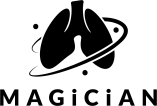


**MAGICIAN** Clinical Trial Protocol

will be done using SAS™ software.

**9.10.**

**Interim analysis**

Given the long study timeline, one interim analysis is planned after we recruit 50% of our sample. We will follow the O’Brien-Fleming group sequential method with a level of significance of 0.0054 at the interim analysis and 0.0492 for the final analysis. This analysis will be conducted by the statistician assigned to the DSMC (not otherwise involved in the trial) and evaluated by the DSMC.

**9.11.**

**Subgroup analyses**

*A priori* we identified the following subgroups for subgroup analyses: age ≤5 vs ≥6 years,53,70 sex,71 post-randomization PRAM score, personal atopy, non-rhinovirus viral nasal pathogen, and “acute viral induced wheeze” phenotype (age ≤5 years without atopy or cough between respiratory infections).72,73 For subgroup analyses, we will use generalized linear mixed modelling with treatment group-subgroup interaction factor, controlling for site and age randomization factors. *Sex subgroup analysis* **–** while we are unable to stratify randomization by sex due to feasibility, sex will be considered in a pre-identified key subgroup analysis.

**9.12.**

**Economic analysis**

We will assess the cost-effectiveness of IVMg to alleviate respiratory distress. We will use cost-utility analysis (CUA) to assess the incremental cost-effectiveness of IVMg vs placebo in terms of cost per quality-adjusted life year and cost-effectiveness analysis to assess cost per alleviating respiratory distress (PRAM ≤3). Using a decision tree, analyses will be undertaken from the perspective of the Canadian healthcare system over a 72-hour horizon which will conform to Canadian guidelines for economic evaluation.74

Comparative effectiveness of IVMg versus placebo will be derived from the MAGICIAN data, including PRAM ≤3 points at 3 hours (secondary outcome c); patient disposition (i.e., discharge or hospitalization during the index ED visit; secondary outcome a), resource use (secondary outcomes d–f), and adverse events. Costs will include drug acquisition and administration, healthcare resource use, and adverse event management. For the CUA, utilities will be sourced from the literature. Scenario analyses will include adopting a societal perspective. Further information can be found in Appendix E: Economic Analysis.

**9.13.**

**Adherence**

This is unlikely to be a problem since intervention delivery will be given in the ED. The experimental period is short and IV administration guarantees medication delivery.

Conservatively, we assume a 5% crossover between groups; the sample size has been adjusted accordingly.

Version Date: June 25, 2025 (v3.0)

Page **28** of **54**


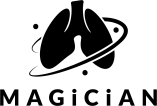


**MAGICIAN** Clinical Trial Protocol

**9.14.**

**Follow up loss**

Due to the short follow-up period, we expect the loss to follow-up to be zero. In MAGNUM with the same follow-up interval, follow-up loss was 1/818. Studies of asthma and bronchiolitis from SickKids had follow up rates at 28 days approaching 100%.75,76 Conservatively, we anticipate the rate of the loss to follow up at 1%, although it will likely be lower.

**10. Adverse Events and Serious Adverse Events**

A Data Safety Monitoring Committee (DSMC) will be created to review this study, detailed in Section 11.2 (Safety Oversight – Data Safety and Monitoring Committee (DSMC)).

**10.1.**

**Definition of Adverse Events (AE)**

An Adverse Event (AE) is any untoward medical occurrence associated with the use of an intervention in a study participant, which does not necessarily have a causal relationship with the intervention. An AE can therefore be any unfavourable and unintended sign (including an abnormal laboratory finding), symptom or disease temporally associated with the use of the intervention, whether or not considered related to the investigational intervention. Stable chronic conditions which are present prior to entry in the study and do not worsen are not considered AE. These pre-existing conditions will be documented in the participant’s medical history.

A qualified physician, who is part of the study team, will be responsible for determining whether an AE is expected or unexpected, and the relationship to the study intervention will be described as unrelated, unlikely to be related, possibly related, probably related, or related, based on the temporal relationship to the administration of the study intervention and the study physician's clinical judgment.

An AE will be considered unexpected if the nature, severity, or frequency of the event is not consistent with the risk information previously described in section

2.3 (Risk/Benefit Assessment - Participant Safety) and Product Monograph for magnesium.

Adverse events will be collected using Common Terminology Criteria for Adverse Events (CTCAE) version 5.0.

**10.2.**

**Definition of Serious Adverse Events (SAE)**

Version Date: June 25, 2025 (v3.0)

Page **29** of **54**


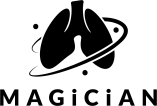


**MAGICIAN** Clinical Trial Protocol

A Serious Adverse Event is any AE that is:

- fatal

•

•

•

•

•

life-threatening

requires or prolongs inpatient hospital stay

results in persistent or significant disability or incapacity a congenital anomaly or birth defect

an important medical event

The term “life-threatening” in the definition of “serious” refers to an AE in which the participant was at risk of death at the time of the event. It does not refer to an AE that hypothetically might have caused death if it were more severe.

Important medical events are those that may not be immediately life threatening but are clearly of major clinical significance. They may jeopardize the participant and may require intervention to prevent one of the other serious outcomes noted above. Examples of such medical events include allergic bronchospasm requiring intensive treatment in an emergency room or at home, blood dyscrasias or convulsions that do not result in inpatient hospitalization, or the development of drug dependency or drug abuse

**Serious Adverse Event (SAE)** will be documented for any incident that consist of:

1. hypotension below the 5th percentile for age requiring medical intervention within 180 minutes of the start of the experimental therapy, or
2. admission to intensive care unit (ICU) at the index ED visit.
3. admission to intensive care unit (ICU) for asthma within 72 hours after being discharged from ED.

These will be reported to the PI, SickKids REB, local REB and the DSMC.

Since hypotension is the only side-effect of IVMg occurring with appreciable frequency, all enrolled patients will be on precautionary frequent blood pressure monitoring as per the study protocol. If the systolic blood pressure drops below 5th percentile for age, treatment will be given as necessary and DSMC will be notified.

**10.3.**

**Classification of an Adverse Event**

**10.3.1. Severity of Event**

The following guidelines will be used to describe severity – as adapted from Common Terminology Criteria for Adverse Events (CTCAE) v5.0.

•

**Grade 1 Mild:** Mild; asymptomatic or mild symptoms; clinical or diagnostic observations only; intervention not indicated.

•

**Grade 2 Moderate:** Moderate; minimal, local or non-invasive

Version Date: June 25, 2025 (v3.0)

Page **30** of **54**


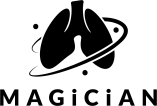


**MAGICIAN** Clinical Trial Protocol

intervention indicated; limiting age-appropriate instrumental activities of daily living (ADL), such as preparing meals, shopping for groceries or clothes, using the telephone, managing money.

•

**Grade 3 Severe:** Severe or medically significant but not immediately life- threatening; hospitalization or prolongation of hospitalization indicated; disabling; limiting self-care ADL, such as bathing, dressing and undressing, feeding self, using the toilet, taking medications, and not bedridden.

•

**Grade 4 Life-threatening:** Life-threatening consequences; urgent intervention indicated.

•

**Grade 5 Death:** Related to AE.

**10.3.2. Relationship to Study Intervention**

All Adverse Events (AEs) must have their relationship to the study intervention assessed by a qualified physician who is part of the study team based on temporal relationship and their clinical judgment. The degree of certainty about causality will be graded using the categories below.

•

**Definitely Related**– There is clear evidence to suggest a causal relationship, and other possible contributing factors can be ruled out. The clinical event, including an abnormal laboratory test result, occurs in a plausible time relationship to study intervention administration and cannot be explained by concurrent disease or other drugs or chemicals. The response to withdrawal of the study intervention should be clinically plausible.

•

**Probably Related** – There is evidence to suggest a causal relationship, and the influence of other factors is unlikely. The clinical event, including an abnormal laboratory test result, occurs within a reasonable time after administration of the study intervention, is unlikely to be attributed to concurrent disease or other drugs or chemicals, and follows a clinically reasonable response on withdrawal.

•

**Possibly Related** – There is some evidence to suggest a causal relationship (e.g., the event occurred within a reasonable time after administration of the study intervention). However, other factors may have contributed to the event (e.g., the participant’s clinical condition, other concomitant events).

•

**Unlikely to be related** – A clinical event, including an abnormal laboratory test result, whose temporal relationship to study intervention administration makes a causal relationship improbable (e.g., the event did not occur within a reasonable time after administration of the study intervention) and in which other drugs or chemicals or underlying disease provides plausible

Version Date: June 25, 2025 (v3.0)

Page **31** of **54**


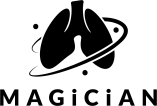


**MAGICIAN** Clinical Trial Protocol

explanations (e.g., the participant’s clinical condition, other concomitant

treatments).

•

**Unrelated** – The AE is completely independent of study intervention administration, and/or evidence exists that the event is definitely related to another etiology.

**10.3.3. Expected adverse events**

Expected adverse events that are known to be related to asthma or accompanying viral illness will be recorded on the case report form but not reported. These will include cough, fever, respiratory distress, nausea/vomiting, diarrhea, sore throat, nasal congestion, supplemental oxygen, use of high-flow nasal cannula, skin rash, diaper rash, asthma-related hospitalization, asthma-related unscheduled medical visits with 72 hours of index presentation, IV insertion, sinus tachycardia.

The product monograph mentions the following as magnesium-related potential adverse events: flushing, sweating, hypotension, circulatory collapse, cardiac and central nervous system depression and respiratory depression.

While flushing and sweating will be considered expected and unreported adverse events, the remaining events, while exceedingly unlikely, mandate ICU admission, and would thus be considered SAEs.

Magnesium blocks the neuromuscular transmission and acts as a CNS depressant. Therefore, the theoretical adverse effects with IV Mg may include a transient drop in blood pressure, apnea and heart block. However, only hypotension has been reported with any frequency, which is low.

**10.3.4. Time Period and Frequency for Event Assessment and Follow-Up**

All Adverse Events (AEs) or Serious Adverse Events (SAEs) with start dates occurring any time after receiving the study intervention until 3 days (for non- serious AEs) or 3 days (for SAEs) after the last day of study intervention will be documented.

Participants who are discharged from the ED will receive a survey via email or phone call 72 hours (3 days) after leaving the ED to gather information on any events since leaving the ED.

Participants who are admitted to the hospital for an SAE will be followed for outcome information until resolution or in the opinion of a qualified physician delegate, the participate is stable and does not require further follow-up. These participants will not receive the survey 72 hours (3 days) later.

**10.3.5. Reporting of Adverse Events**

Version Date: June 25, 2025 (v3.0)

Page **32** of **54**


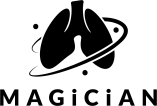


**MAGICIAN** Clinical Trial Protocol

All **unexpected adverse events** will be reported to The Hospital for Sick Children Research Ethics Board according to The Hospital for Sick Children’s Adverse Event Reporting requirements and as per the participating site’s local institutional and regulatory requirements.

All serious, unexpected adverse drug reactions to the study medication will be reported to Health Canada within 15 calendar days or for death or life-threatening events, within 7 calendar days. In the latter case, a follow-up report must be filed within 8 calendar days. Adverse reactions will be managed according to the local practices of participating site’s.

**10.3.6. Serious Adverse Event Reporting**

All Serious Adverse Events (SAE) must be reported to Dr. Suzanne Schuh within 24 hours of becoming aware of the SAE. The initial report must be emailed to [Suzanne.schuh@sickkids.ca.](mailto:Suzanne.schuh@sickkids.ca) and should contain as much information as available. At a minimum, the report must contain:

•

•

•

•

Name of Site and Principal Investigator, Participant Identification Code, Adverse Event Term,

Study Drug Dose and Start/Stop Dates

Dr. Schuh will assess the event and confirm if it meets Health Canada’s reporting

requirements.

**Only adverse drug reactions that are both serious and unexpected are subject to expedited reporting to Health Canada**. Expedited reporting of reactions which are serious but expected is not required. Expedited reporting is also inappropriate for serious events from clinical investigations that are considered unrelated to the study product, whether or not the event is expected.

AE will be reported in accordance with site REB and regulatory authorities in accordance with local institutional and regulatory requirements.

Adverse events will be reported to The Hospital for Sick Children through the REDCap database.

Dr. Suzanne Schuh will notify all Investigators of all Serious Adverse Events that are reportable to regulatory authorities in Canada from this trial as described above. Investigators must notify their Research Ethics Boards (REBs) according to institutional requirements and file the report and acknowledgement from the REB (e.g. letter from the REB acknowledging receipt, stamp from the REB, signed and dated by REB chair or delegate, acknowledging receipt) with their

Version Date: June 25, 2025 (v3.0)

Page **33** of **54**


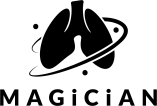


**MAGICIAN** Clinical Trial Protocol

Investigator Site File.

The Data Safety Monitoring Committee (DSMC) will be notified by email of all unexpected Adverse Events.

**11. SUPPORTING DOCUMENTATION AND OPERATIONAL CONSIDERATIONS**

**11.1.**

**Confidentiality and Privacy**

Participant confidentiality and privacy is strictly held in trust by the participating Investigators, their staff, and the Sponsor(s). This confidentiality is extended to cover the clinical information relating to participants. Therefore, the study protocol, documentation, data, and all other information generated will be held in strict confidence.

Any research information obtained about the patient in this study will be kept confidential. A patient will not be identified by name, only by unique study ID number. The patient’s name or any identifying information will not appear in any reports published as a result of this study. All identifying information will be kept behind 2 security measures or as per equivalent institutional policy, under the supervision of the study/site PI and will not be transferred outside of the hospital.

The study monitor, auditor and other authorized representatives of the Sponsor, representatives of the Research Ethics Board (REB), Governors of the University of Alberta, as represented by the Women & Children’s Health Research Institute, and regulatory agencies may inspect all documents and records required to be maintained by the Investigator, including but not limited to, medical records and pharmacy records for the participants in this study. The clinical study site will permit access to such records.

Study participant research data, which is for purposes of statistical analysis and scientific reporting, will be transmitted to and stored at the Governors of the University of Alberta, as represented by the Women & Children’s Health Research Institute. This will not include the participant’s contact or identifying information. Rather, individual participants and their research data will be identified by a unique study identification number. The study data entry and study management systems used by clinical sites and by Governors of the University of Alberta, as represented by the Women & Children’s Health Research Institute research staff will be secured and password protected. At the end of the study, all study databases will be de-identified and archived at the The Hospital for Sick Children.

**11.2.**

**Safety Oversight - Data Safety and Monitoring Committee (DSMC)**

Safety oversight will be under the direction of the Data Safety and Monitoring Committee (DSMC). The DSMC members will be listed in the DSMC charter.. The members of this committee will not be collaborators of this trial. They will be notified of all serious adverse events and provide feedback to the Trial Steering Committee to determine if the study should be stopped early, continued or terminated. A DSMC

Version Date: June 25, 2025 (v3.0)

Page **34** of **54**


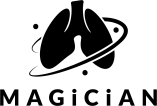


**MAGICIAN** Clinical Trial Protocol

charter will be developed in conjunction with the DSMC members to guide the committee process. The DSMC will meet every 6 months or ad hoc if necessary.

**11.3.**

**Participant Safety**

Magnesium has a theoretical potential for hypotension, hypopnea and heart block,45 but only hypotension has been reported in previous studies. The IVMg RCT of 1109 adults reported 8% hypotension46 but nobody had Mg stopped for hypotension.

Pediatric IVMg trials with Mg doses 75 and 100 mg/kg did not report any hypotension (N=101).15,20 While a review of the PECARN Registry found a median dose 50 mg/kg had a hypotension rate of 6.8%,10 the mean decrease in the systolic pressure was 5 mm Hg, with uncertain clinical significance. No hypotension was observed in 3 pediatric pharmacologic ICU studies of 75 mg/kg IV Mg in children

≤30 kg and 50 mg/kg in those >30 kg followed by 40 mg/kg/hour for 4 hours.47-49 Two of our participating sites routinely use 75 mg/kg IVMg. A literature review of pediatric status asthmaticus confirms lack of IVMg toxicity.50 Nonetheless, blood pressure will be measured at 30 and 60 minutes and hourly to 180 minutes. If the systolic pressure drops below the 5th percentile-for-age,51 necessary treatment such as IV fluids will be given. If the hypotension occurs at either the 10 minute or 20 minute measurement, further experimental infusion will be stopped and blood pressure will be treated according to local practices. Hypotension will be defined as systolic blood pressure

<70 mm Hg +(age in years x2) in children 2-10 years old and as <90 mm Hg in those >10 years old. Because the children with unstable airway will be excluded and further inhaled salbutamol will be given if needed, lack of Mg in the placebo group will not endanger these participants. All study patients will be monitored for 180 minutes post-intervention prior to discharge, to ensure safety. Only13/723(1.8%) children discharged post IVMg return to the ED vs 1383/38,623(3.6%) without; IVMg is not associated with re-visits.10

**11.4.**

**Study Monitoring**

Monitoring of the trial will be performed to verify that:

- The rights and well-being of participants are protected;

•

The reported trial data are accurate, complete, and verifiable from source documents; and

The conduct of the trial is in compliance with the currently approved protocol/amendment(s), ICH GCP, and local regulations and requirements.

•

The Sponsor will be responsible for all monitoring activities. Any trial-related duty or function transferred to and assumed by a third party, including monitoring and auditing, will be specified in a clinical trial agreement and oversight provided by the Sponsor.

The monitoring plan for the trial will be documented prior to the activation of the study and include the following;

•

Follow risk-based practices,

Version Date: June 25, 2025 (v3.0)

Page **35** of **54**


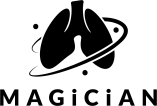


**MAGICIAN** Clinical Trial Protocol

•

•

Document the rationale for the chosen monitoring strategy,

Reference the Sponsor’s process that will be followed to address situations of

non-compliance,

Describe the monitoring responsibilities of all the parties involved, and Outline the data and processes to be monitored.

•

•

The site Investigator(s)/delegate(s) will allow direct access to source data/documents for the purposes of monitoring by the Sponsor, and inspection by regulatory authorities, both domestic and foreign (if applicable). It is important that the Sponsor, site Investigator and site personnel are available during monitoring visits and inspections, and that sufficient time is devoted to the process.

Monitoring procedures will be implemented beginning with the data entry system and data checks that will be run on the database will be generated. Any missing data or data anomalies will be communicated to the site(s) for clarification/resolution.

Monitoring reports will be issued after each monitoring visit for review and follow up by the Sponsor, site Investigator, and appropriate management and personnel responsible for trial and site oversight.

**11.5.**

**Quality Assurance and Quality Control**

Each site should have SOPs for quality management that describe:

- The documents to be reviewed (e.g., CRFs, clinic notes, product accountability records, etc), who is responsible, and the frequency for reviews.

•

Who will be responsible for addressing QA issues (e.g., correcting procedures that are not in compliance with protocol) and QC issues (e.g., correcting errors in data entry).

Staff training methods and how such training will be tracked.

•

Regular monitoring and an independent audit, if conducted, must be performed according to ICH GCP. See also Section 11.4 (Study Monitoring).

Each clinical site will perform internal quality management of study conduct, data, documentation and completion.

Auditing of the trial will be performed independently from monitoring to evaluate trial conduct and compliance with the protocol/amendment(s), SOP, ICH GCP and local regulations and requirements.

The Sponsor will be responsible for all auditing activities. Any trial-related duty or function transferred to and assumed by a third party, including auditing, will be specified in a clinical trial agreement and oversight provided by the Sponsor.

Version Date: June 25, 2025 (v3.0)

Page **36** of **54**


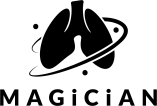


**MAGICIAN** Clinical Trial Protocol

**11.6.**

**Data Handling and Record Keeping**

**11.6.1. Data Collection and Management Responsibilities**

Data collection is the responsibility of the clinical trial staff at the site under the supervision of the site Investigator. The Investigator is responsible for ensuring the accuracy, completeness, legibility, and timeliness of the data reported.

All source documents should be completed in a neat, legible manner to ensure accurate interpretation of data.

Where the source data is not collected as part of the participant’s medical record, hardcopies of the study visit worksheets will be provided for use as source document worksheets for recording data for each participant enrolled in the study. Data recorded in the case report form (CRF) derived from source documents should be consistent with the data recorded on the source documents.

Study data will be entered into REDCap (Research Electronic Data Capture), a secure, web-based application designed exclusively to support data capture for research studies. REDCap is a web-based electronic data capture system and it is licensed by the University of Alberta and hosted in a secure server environment provided by the Faculty of Medicine.

**11.6.2. Study Records Retention**

To enable evaluations and/or audits from Health Canada and/or the Sponsor, the Principal Investigator agrees to keep records, including the identity of all participating patients (sufficient information to link records, CRFs and hospital records), all original signed informed consent forms, copies of all CRFs, source documents, and detailed records of treatment disposition in a secure location for a minimum of 15 years.

If the Principal Investigator relocates, retires, or for any reason withdraws from the study, then the Sponsor should be prospectively notified. The study records must be transferred to an acceptable designee, such as another Investigator, another institution, or to the Sponsor.

**11.7.**

**Protocol Deviations**

A protocol deviation is any noncompliance with the clinical trial protocol or Manual of Operations (MOO) requirements, if applicable. The Principal Investigator will assure that both the Sponsor and the REB will be notified of protocol deviations in accordance with Sponsor and local REB requirements. The noncompliance may be either on the part of the participant, the Investigator, or the study site staff. Depending on the type of protocol

Version Date: June 25, 2025 (v3.0)

Page **37** of **54**


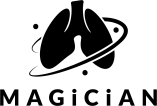


The results of this study will be submitted for presentation at a scientific conference and the manuscript will be submitted for publication in a peer-reviewed scientific journal.

**MAGICIAN** Clinical Trial Protocol

deviations, sites may be required to develop and implement corrective and preventative actions . All protocol deviations will be documented using a protocol deviation log; the Principal Investigator will assess each protocol deviation to determine the impact to the patient’s rights, safety or welfare, study efficacy and data integrity. If there is any uncertainty regarding the impact of the protocol deviation, the Principal Investigator will consult with Dr. Suzanne Schuh.

**11.8.**

**Publication and Dissemination of Results**

This study will comply with the CIHR Open Access Policy. The study has been registered at ClinicalTrials.gov (NCT06785272), and results information from this study will be submitted to ClinicalTrials.gov. Conduct, reporting, editing, and publication of resultant scholarly work will be guided by the International Committee of Journal Medical Editors (ICJME)’s published recommendations. The identity of participants will not be revealed in any published data or in presentation of the information obtained for this study.

Version Date: June 25, 2025 (v3.0)

Page **38** of **54**


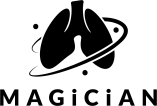


**MAGICIAN** Clinical Trial Protocol

**APPENDIX A - Abstract from our MAGNUM RCT**

**Effect of Nebulized Magnesium vs Placebo Added to Albuterol on Hospitalization Among Children with Refractory Acute Asthma Treated in the Emergency Department-A Randomized Clinical Trial**

Suzanne Schuh, MD; Judy Sweeney, RN, BScN; Maggie Rumantir, MD; Allan L. Coates, MDCM, BEng; Andrew R. Willan, PhD; Derek Stephens, MSc, BSc; Eshetu G. Atenafu, MSc; Yaron Finkelstein, MD; Graham Thompson, MD; Roger Zemek, MD; Amy C. Plint, MD, MSc; Jocelyn Gravel, MD, MSc; Francine M. Ducharme, MD, MSc; David W. Johnson, MD; Karen Black, MD, MSc; Sarah Curtis, MD; Darcy Beer, MD; Terry P. Klassen, MD, MSc; Darcy Nicksy, BSc, PhM; Stephen B. Freedman, MDCM, MSc; for the Pediatric Emergency Research Canada (PERC) Network

***JAMA*. 2020**;324(20):2038-2047. doi:10.1001/jama.2020.19839

**Importance:** While intravenous magnesium decreases hospitalizations in refractory pediatric acute asthma, it is variably used because of invasiveness and safety concerns. The benefit of nebulized magnesium to prevent hospitalization is unknown.

**Objective:** To evaluate the effectiveness of nebulized magnesium in children with acute asthma remaining in moderate or severe respiratory distress after initial therapy.

**Design, Setting and Participants:** A randomized double-blind parallel-group clinical trial from September 26, 2011, to November 19, 2019, in 7 tertiary-care pediatric emergency departments in Canada. The participants were otherwise healthy children aged 2 to 17 years with moderate to severe asthma defined by a Pediatric Respiratory Assessment Measure (PRAM) score of 5 or greater (on a 12-point scale) after a 1-hour treatment with an oral corticosteroid and 3 inhaled albuterol and ipratropium treatments. Of 5846 screened

patients, 4332 were excluded for criteria, 273 declined participation, 423 otherwise excluded, 818 randomized, and 816 analyzed.

**Interventions:** Participants were randomized to 3 nebulized albuterol treatments with either magnesium sulfate (n = 410) or 5.5%saline placebo (n = 408).

MAIN OUTCOMES AND MEASURES The primary outcome was hospitalization for asthma within

24 hours. Secondary outcomes included PRAM score; respiratory rate; oxygen saturation at 60, 120, 180, and 240 minutes; blood pressure at 20, 40, 60, 120, 180, and 240 minutes; and albuterol treatments within 240 minutes.

**Results:** Among 818 randomized patients (median age, 5 years; 63%males), 816 completed the trial (409 received magnesium; 407placebo). A total of 178 of the 409 children who received magnesium (43.5%) were hospitalized vs 194 of the 407 who received placebo (47.7%) (difference, −4.2%; absolute risk difference 95%[exact] CI, −11% to 2.8%]; *P* = .26).

There were no significant between-group differences in changes from baseline to 240

minutes in PRAM score (difference of changes, 0.14 points [95% CI, −0.23 to 0.50]; *P* = .46);

respiratory rate (0.17 breaths/min [95%CI, −1.32 to 1.67]; *P* = .82); oxygen saturation

Version Date: June 25, 2025 (v3.0)

Page **39** of **54**


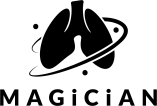


**MAGICIAN** Clinical Trial Protocol

(−0.04% [95%CI, −0.53%to 0.46%]; *P* = .88); systolic blood pressure (0.78mmHg [95%

CI, −1.48 to 3.03]; *P* = .50); or mean number of additional albuterol treatments (magnesium: 1.49, placebo: 1.59; risk ratio, 0.94 [95%CI, 0.79 to 1.11]; *P* = .47). Nausea/vomiting or sore throat/nose occurred in 17 of the 409 children who received magnesium (4%) and 5 of the 407 who received placebo (1%).

**Conclusions and Relevance:** Among children with refractory acute asthma in the emergency department, nebulized magnesium with albuterol, compared with placebo with

albuterol did not significantly decrease the hospitalization rate for asthma within 24 hours. The findings do not support use of nebulized magnesium with albuterol among children with refractory acute asthma.

Version Date: June 25, 2025 (v3.0)

Page **40** of **54**


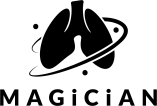


**MAGICIAN** Clinical Trial Protocol

**APPENDIX B - Pediatric Respiratory Assessment Measure (PRAM)a score**

a PRAM score represents the sum of the individual components (range 0-12).25

b Because Calgary is 1000 meters above sea level, the saturation cut-offs have been adjusted accordingly.77

Version Date: June 25, 2025 (v3.0)

Page **41** of **54**

**Signs**

**0**

**1**

**2**

**3**

Suprasternal retractions

Absent

Present

Scalene muscle contraction

Absent

Present

Air entry

Normal

Decreased at bases

Widespread decrease

Absent/minimal

Wheezing

Absent

Expiratory only

Inspiratory and expiratory

Audible without stethoscope/ silent chest with minimal air entry

O2 saturation

≥95%

92%-94%

≤91%

O2 saturation Calgaryb

≥93%

90%-92%

≤89%


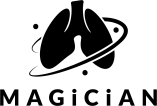


**MAGICIAN** Clinical Trial Protocol

**APPENDIX C - Abstract from our secondary MAGNUM analysis**

**Association between Intravenous Magnesium and Hospitalization from Emergency Departments in Pediatric Refractory Acute Asthma**

Suzanne Schuh, MD; Stephen B. Freedman, MDCM, MSc; Roger Zemek, MD; Amy C. Plint, MD, MSc; David W. Johnson, MD; Francine Ducharme, MD, MSc; Jocelyn Gravel, MD, MSc; Graham Thompson, MD; Sarah Curtis, MD; Derek Stephens, MSc, BSc; Allan L. Coates, MDCM, BEng; Karen J Black, MD, MSc; Darcy Beer, MD; Judy Sweeney, RN, BScN; Maggie Rumantir, MD; Yaron Finkelstein, MD; for the Pediatric Emergency Research Canada (PERC) Network

**JAMA Network Open, 2021**

**Importance:** Despite the guideline recommendation of IV magnesium to reduce hospitalizations in refractory pediatric acute asthma and increasing IV magnesium use, asthma-related hospitalizations have remained stable. Emergency department (ED) physicians may hesitate to discharge children home after IV magnesium.

**Objective:** To determine the association between IV magnesium therapy in the ED and hospitalization, after adjustment for patient-level variables. We hypothesized that children given IV magnesium would be at higher likelihood of hospitalization.

**Design:** Prospective cohort study representing a planned secondary analysis of a double-blind clinical trial of children with acute asthma randomized to three nebulized treatments of albuterol plus either magnesium sulfate or 5.5% saline placebo. Thereafter, IV magnesium therapy was used as per ED physician decision, representing the intervention of interest in the current study.

**Setting:** Seven Canadian tertiary-care pediatric EDs.

**Participants:** 816 otherwise healthy children 2-17 years old with Pediatric Respiratory Assessment Measure (PRAM) ≥5/12 points after initial therapy with systemic corticosteroids and inhaled albuterol with ipratropium.

**Exposure: IV magnesium sulfate (40-50 mg/kg) after inhaled magnesium/placebo.**

**Main Outcome(s) and Measure(s):** Hospitalization for asthma from the ED. Candidate predictor variables included year-epoch at enrollment, IV magnesium, PRAM after initial therapy, PRAM at ED disposition, age, sex, respiratory distress duration, previous ICU asthma admission, asthma hospitalizations within the past year, atopy, oral corticosteroids within 48 hours before arrival, nebulized magnesium, and additional albuterol after inhaled magnesium/placebo, with site as a random effect.

Version Date: June 25, 2025 (v3.0)

Page **42** of **54**


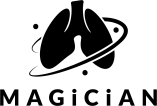


**MAGICIAN** Clinical Trial Protocol

**Results:** 364/816 (44.6%) patients were hospitalized, 215/816 (26.3%) received IV magnesium; 190/215 (88.4%) children given IV magnesium were hospitalized. Multivariable predictors of hospitalization were IV magnesium in 2011-2016 [OR 22.67(95% CI 6.26-82.06) p<0.001] and in 2017-2019 [OR 4.19 (1.99-8.86) p<0.001], additional albuterol [OR 5.53(3.27-9.37) p<0.001]

and disposition PRAM [OR 2.28(1.92-2.71) p<0.001].

**Conclusions and Relevance:** After adjustment for patient-level characteristics, IV magnesium after initial ED asthma treatment is associated with hospitalization. Definitive evidence of IV magnesium benefit on hospitalization would clarify its role in refractory pediatric asthma.

Version Date: June 25, 2025 (v3.0)

Page **43** of **54**


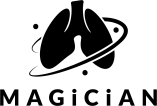


**MAGICIAN** Clinical Trial Protocol

**APPENDIX D - Abstract from our international PERN survey**

**International Practice Patterns in Intravenous Magnesium Therapy in Pediatric Refractory Acute Asthma**

Laura Simone MD, MSc(c), FRCPC, Roger Zemek, MD; Damian Roland, MD, BMBS; Mark D Lyttle, MBChB; Simon Craig; Stuart R Dalziel, MBChB, FRACP, PhD; Jocelyn Gravel; Yaron Finkelstein, MD; Sarah Curtis, MD; Stephen B. Freedman, MDCM, MSc; Amy C. Plint , MD MSc; Naveen Poonai, MD, MSc; Franz E Babl, MD, MPH; Graham Thompson, MD, MSc; Francine M Ducharme, MD, MSc; David W. Johnson, MD; Terry P Klassen, MD, MSc; Bethany Lerman; Suzanne Schuh, MD for the Pediatric Emergency Research Canada (PERN) Network

**Emergency Medicine Journal, 2022**

**Background and Objectives**

Guidelines recommend intravenous magnesium (IV-Mg) in pediatric severe refractory asthma, despite limited evidence of benefit. The primary objective was to determine the proportion of physicians agreeing there is adequate evidence that children with sustained satisfactory response to IV-Mg in the emergency department can be safely discharged.

**Methods**

We conducted a cross-sectional survey of pediatric emergency physicians at research networks in Canada, Australia/New Zealand and the U.K./Ireland (March-June 2021), using a modified Dillman technique. Two clinical vignettes, describing a case of severe refractory asthma, preceded the survey. After bronchodilator and corticosteroid therapy, asthma severity in vignette 1 does not change and becomes moderate in vignette 2.

**Results**

657/886 (74%) physicians responded; 586 were eligible.104/586 (17.7%) participants stated that evidence regarding the safety of discharging patients with satisfactory response to IV-Mg is sufficient to support adoption. The goal of IV-Mg to decrease hospitalization in Canada vs elsewhere was expressed by 52.7% vs 3.3% participants in vignette 1 [OR 32.3 (17.1-64.7] and

51.9% vs 4.0% in vignette 2 [OR 50.4 (24.3-115.6]. A total of 443/586 (76%) physicans use IV- Mg to provide respiratory distress relief. 488 respondents (83.3%) stated that prescribing IV-Mg also prompts them to hospitalize, irrespective of clinical response. 455/586 participants (77.6%) agree that a definitive trial is needed to clarify IV-Mg benefit and 80% indicate that evidence of IV Mg benefit would change their practice.

**Conclusions**

A minority of physicians believe there is adequate evidence for IV-Mg benefit. International variability exists in physician acceptability to discharge following a sustained satisfactory response to IV-Mg. Conclusive evidence of IV-Mg benefit would inform the role of IV-Mg in refractory pediatric asthma and clarify the safety of discharge.

Version Date: June 25, 2025 (v3.0)

Page **44** of **54**


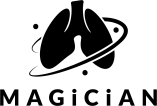


**MAGICIAN** Clinical Trial Protocol

**APPENDIX E - Economic analysis**

The cost-effectiveness of IVMg versus placebo to alleviate respiratory distress will be assessed from the perspective of the Canadian public health care system. To this end, trial-based cost- utility analysis (CUA) and cost-effectiveness analyses (CEA) will be conducted, using a decision-tree framework. First, CUA will be performed, in which costs and quality-adjusted life years (QALYs) associated with each treatment will be estimated, and the incremental cost per QALY gained will be determined. Second, a CEA will be used to assess the incremental cost per alleviating respiratory distress (i.e., achieving mild asthma status indicated by PRAM ≤ 3 points). Although the MCID for PRAM in the MAGICIAN trial is a change of at least 1 point, the decision problem will focus on the alleviation of respiratory distress (PRAM ≤ 3 points) as signifies dischargable mild asthma. Both analyses will be conducted over a 72-hour horizon, given that all costs and benefits of IVMg treatment are expected to be experienced within 72 hours. In the CUA, the accumulated QALYs for both treatments are expected to be small owing to the 72-hour time frame; however, the cost-effectiveness of IVMg will be informed by the incremental difference in QALYs (as part of the incremental cost-utility ratio calculation). All analyses will adhere to the Guidelines for the Economic Evaluation of Health Technologies: Canada (CADTH 2017; 4th edition).

Comparative effectiveness of IVMg versus placebo will be derived from the MAGICIAN trial. Treatment response (PRAM indicating mild asthma [≤3 points]; secondary outcome c) will be assessed at 180 minutes. Patient disposition (i.e., discharge or admission to hospital for asthma) during the index ED visit; secondary outcome a), resource use (secondary outcomes d, e, f), and adverse events will additionally be obtained from the MAGICIAN trial. To determine costs, a unit cost will be applied to each type of resource, and the total cost will be estimated as the weighted sum of resource use (the sum of the product of the number of each resource item and its unit cost). Costs associated with AEs will be based on the observed frequency of each AE in the MAGICIAN trial and costs associated with their management. The resources, type of measurement, and source of the cost data are described in the following table:

For the CUA, utilities will be incorporated for treatment response (mild asthma) or no treatment response (moderate-to-severe asthma) and disutilities will be included for adverse events (e.g., hypotension). Utilities and disutilities will be obtained from the literature via a systematic search, with the choice of values based on their fitness for purpose, credibility, and consistency.

Probabilistic analyses will be used to incorporate uncertainty around the estimate of costs and outcomes. Discounting will not be applied, as the horizon is less than one year. Results will be displayed via a cost-effectiveness acceptability curve, and disaggregated costs and benefits will

Version Date: June 25, 2025 (v3.0)

Page **45** of **54**

**Resource item**

**Source of cost**

Drug acquisition

Hospital pharmacy

Drug administration

Provincial fee schedule (Ministry of Health and Long-Term Care)

Hospitalization

Provincial ministry (Ontario Case Costing Initiative)

Physician consultation

Provincial fee schedule (Ministry of Health and Long-Term Care)

Emergency department visit

Provincial fee schedule (Ministry of Health and Long-Term Care)


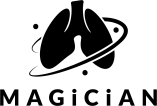


**MAGICIAN** Clinical Trial Protocol

be reported. Scenario and sensitivity analyses will be undertaken to explore the impact of uncertainty within the analyses (e.g., structural or parameter uncertainty).

Version Date: June 25, 2025 (v3.0)

Page **46** of **54**


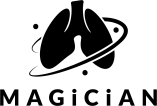


**MAGICIAN** Clinical Trial Protocol

**APPENDIX F - Annual Enrollment Projection**

**+** Informed by MAGNUM schedule of 12 hours/day weekdays and 12 hours 1 weekend day a week; 10% miss rate, screens represent approximately 30% of annual presentations

***** Projected randomizations are conservatively estimated on 75% of screened children excluded for criteria, and approx 50% of eligible patients not participating due to refusals/non- participation for other reasons (based on MAGNUM).

****** Numbers with full data are conservatively based on 5% rate of non-compliance with allocated therapy and 1% loss to follow-up (1/818 in MAGNUM).

Version Date: June 25, 2025 (v3.0)

Page **47** of **54**

**Annual ED Asthma Presentations**

**≥2 years old**

**Projected Annual Screens+**

**Randomizations***

**Projected Annual Study Numbers with Full Data ****

**HSC**

900

300

50

45

**CHEO**

900

200

14

12

**Alberta Children’s**

750

200

33

30

**Stollery**

300

100

6

5

**St Justine**

750

200

14

12

**McMaster**

300

100

6

5

**Total**

**3900**

**1100**

**123**

**109**


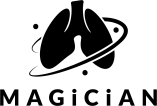


**MAGICIAN** Clinical Trial Protocol

**References**

1.

Mannino DM, Homa DM, Pertowski CA, et al. Surveillance for asthma--United States, 1960-1995. MMWR CDC Surveill Summ 1998;47(1):1-27.

2.

Weiss AJ, Wier LM, Stocks C, Blanchard J. Overview of Emergency Department Visits in the United States, 2011. Healthcare Cost and Utilization Project (HCUP) Statistical Briefs. Rockville (MD): Agency for Healthcare Research and Quality (US); 2006.

3.

Schuh S, Sweeney J, Rumantir M, et al. Effect of Nebulized Magnesium vs Placebo Added to Albuterol on Hospitalization Among Children With Refractory Acute Asthma Treated in the Emergency Department: A Randomized Clinical Trial. Jama 2020;324(20):2038-2047.

4.

(GINA) GIfA. Global strategy for asthma management and prevention. 2021. (<https://ginasthma.org/wp->content/uploads/2021/2005/GINA-Main-Report-2021-V2022- WMS.pdf.).

5.

National AE PP, Third Expert Panel on the Diagnosis and Management of Asthma. Expert Panel Report 3 (EPR-3): Guidelines for the Diagnosis and Management of Asthma. Bethesda (MD): National Heart, Lung, and Blood Institute (US). 2007.

6.

Ortiz-Alvarez O, Mikrogianakis A. Managing the paediatric patient with an acute asthma exacerbation. Paediatr Child Health 2012;17(5):251-62.

7.

SIGN S. Bronchiolitis in children: a national clinical guideline. 2006.

8.

Scotland HI. BTS/SIGN British guideline on the management of asthma: Quick Reference Guide. A national clinical guideline. 2019. ([https://wwwbrit-](https://wwwbrit-thoracicorguk/document-) [thoracicorguk/document-](https://wwwbrit-thoracicorguk/document-) library/guidelines/asthma/btssign-asthma-guideline-quick- reference-guide-2019/).

9.

(AAH) AAH. Australian Asthma Handbook - Diagnosis and Management. 2020. (<https://www.asthmahandbook.org.au/>).

10.

Johnson MD, Zorc JJ, Nelson DS, et al. Intravenous Magnesium in Asthma Pharmacotherapy: Variability in Use in the PECARN Registry. J Pediatr 2020;220:165- 174.e2.

11.

Arnold DH, Gong W, Antoon JW, et al. Prospective Observational Study of Clinical Outcomes After Intravenous Magnesium for Moderate and Severe Acute Asthma Exacerbations in Children. J Allergy Clin Immunol Pract 2022;10(5):1238-1246.

12.

Tanverdi MS, Navanandan N. Intravenous Magnesium for Acute Asthma Exacerbations in Children: To Mag or Not to Mag. J Allergy Clin Immunol Pract 2022;10(5):1247- 1248.

Version Date: June 25, 2025 (v3.0)

Page **48** of **54**


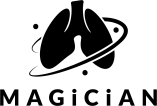


**MAGICIAN** Clinical Trial Protocol

13.

Ciarallo L, Sauer AH, Shannon MW. Intravenous magnesium therapy for moderate to severe pediatric asthma: results of a randomized, placebo-controlled trial. J Pediatr 1996;129(6):809-14.

14.

Ciarallo L, Brousseau D, Reinert S. Higher-dose intravenous magnesium therapy for children with moderate to severe acute asthma. Arch Pediatr Adolesc Med 2000;154(10):979-83.

15.

Scarfone RJ, Loiselle JM, Joffe MD, et al. A randomized trial of magnesium in the emergency department treatment of children with asthma. Ann Emerg Med 2000;36(6):572-8.

16.

Griffiths B, Kew KM. Intravenous magnesium sulfate for treating children with acute asthma in the emergency department. Cochrane Database Syst Rev 2016;4(4):Cd011050.

17.

Liu X, Yu T, Rower JE, Campbell SC, Sherwin CM, Johnson MD. Optimizing the use of intravenous magnesium sulfate for acute asthma treatment in children. Pediatr Pulmonol 2016;51(12):1414-1421.

18.

Antoon JW, Hall M, Mittal V, et al. Intravenous Magnesium and Hospital Outcomes in Children Hospitalized With Asthma. Hosp Pediatr 2021;11(8):785-793.

19.

Mittal V, Hall M, Antoon J, et al. Trends in Intravenous Magnesium Use and Outcomes for Status Asthmaticus in Children's Hospitals from 2010 to 2017. J Hosp Med 2020;15(7):403-406.

20.

Devi PR, Kumar L, Singhi SC, Prasad R, Singh M. Intravenous magnesium sulfate in acute severe asthma not responding to conventional therapy. Indian Pediatr 1997;34(5):389-97.

21.

Gürkan F, Haspolat K, Boşnak M, Dikici B, Derman O, Ece A. Intravenous magnesium sulphate in the management of moderate to severe acute asthmatic children nonresponding to conventional therapy. Eur J Emerg Med 1999;6(3):201-5.

22.

Singhi S, Grover S, Bansal A, Chopra K. Randomised comparison of intravenous magnesium sulphate, terbutaline and aminophylline for children with acute severe asthma. Acta Paediatr 2014;103(12):1301-6.

23.

Schuh S, Freedman SB, Zemek R, et al. Association Between Intravenous Magnesium Therapy in the Emergency Department and Subsequent Hospitalization Among Pediatric Patients With Refractory Acute Asthma: Secondary Analysis of a Randomized Clinical Trial. JAMA Netw Open 2021;4(7):e2117542.

24.

Simone L, Zemek R, Roland D, et al. International practice patterns of IV magnesium in paediatric acute asthma. Emerg Med J 2023;40(3):200-201.

Version Date: June 25, 2025 (v3.0)

Page **49** of **54**


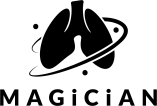


**MAGICIAN** Clinical Trial Protocol

25.

Ducharme FM, Chalut D, Plotnick L, et al. The Pediatric Respiratory Assessment Measure: a valid clinical score for assessing acute asthma severity from toddlers to teenagers. J Pediatr 2008;152(4):476-80, 480.e1.

26.

Lougheed MD, Lemiere C, Ducharme FM, et al. Canadian Thoracic Society 2012 guideline update: diagnosis and management of asthma in preschoolers, children and adults. Can Respir J 2012;19(2):127-64.

27.

Hall IP. Pharmacogenetics of asthma. Eur Respir J 2000;15(3):449-51.

28.

Weiss KB, Sullivan SD, Lyttle CS. Trends in the cost of illness for asthma in the United States, 1985-1994. J Allergy Clin Immunol 2000;106(3):493-9.

29.

Bahadori K, Doyle-Waters MM, Marra C, et al. Economic burden of asthma: a systematic review. BMC Pulm Med 2009;9:24.

30.

Middleton E, Jr. Antiasthmatic drug therapy and calcium ions: review of pathogenesis and role of calcium. J Pharm Sci 1980;69(2):243-51.

31.

Hill J, Britton J. Dose-response relationship and time-course of the effect of inhaled magnesium sulphate on airflow in normal and asthmatic subjects. Br J Clin Pharmacol 1995;40(6):539-44.

32.

Iseri LT, French JH. Magnesium: nature's physiologic calcium blocker. Am Heart J 1984;108(1):188-93.

33.

Classen H JR, Schimatschek H. Interactions of Magnesium with Direct and Indirect Acting Sympathomimetic Amines. Magnesium-Bulletin 1987;9(2):80-87.

34.

Rabe KF, Adachi M, Lai CK, et al. Worldwide severity and control of asthma in children and adults: the global asthma insights and reality surveys. J Allergy Clin Immunol 2004;114(1):40-7.

35.

Rower JE, Liu X, Yu T, Mundorff M, Sherwin CM, Johnson MD. Clinical pharmacokinetics of magnesium sulfate in the treatment of children with severe acute asthma. Eur J Clin Pharmacol 2017;73(3):325-331.

36.

Merckx J, Ducharme FM, Martineau C, et al. Respiratory Viruses and Treatment Failure in Children With Asthma Exacerbation. Pediatrics 2018;142(1)

37.

Sawatzky JA, Enns CL. Exploring the key predictors of retention in emergency nurses. J Nurs Manag 2012;20(5):696-707.

38.

Yazıcı MU, Teksam O, Agın H, et al. The Burden of Burnout Syndrome in Pediatric

Intensive Care Unit and Pediatric Emergency Department: A Multicenter Evaluation.

Version Date: June 25, 2025 (v3.0)

Page **50** of **54**


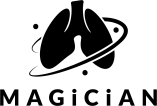


**MAGICIAN** Clinical Trial Protocol

Pediatr Emerg Care 2021;37(12):e955-e961.

39.

Schuh S, Macias C, Freedman SB, et al. North American practice patterns of intravenous magnesium therapy in severe acute asthma in children. Acad Emerg Med 2010;17(11):1189-96.

40.

Taddio A, Soin HK, Schuh S, Koren G, Scolnik D. Liposomal lidocaine to improve procedural success rates and reduce procedural pain among children: a randomized controlled trial. Cmaj 2005;172(13):1691-5.

41.

Arts SE, Abu-Saad HH, Champion GD, et al. Age-related response to lidocaine- prilocaine (EMLA) emulsion and effect of music distraction on the pain of intravenous cannulation. Pediatrics 1994;93(5):797-801.

42.

Manner T, Kanto J, Iisalo E, Lindberg R, Viinamäki O, Scheinin M. Reduction of pain at venous cannulation in children with a eutectic mixture of lidocaine and prilocaine (EMLA cream): comparison with placebo cream and no local premedication. Acta Anaesthesiol Scand 1987;31(8):735-9.

43.

Taddio A, Gurguis MG, Koren G. Lidocaine-prilocaine cream versus tetracaine gel for procedural pain in children. Ann Pharmacother 2002;36(4):687-92.

Schechter NL BC, Yaster M. Pain in infants, children, and adolescents: Lippincott Williams & Wilkins, 2003.

44.

45.

Reynolds J PK, Swetman S, Parsons A. Martindale: The Extra Pharmacopoeia 31st ed: Royal Pharmaceutical Society, London, 1996.

46.

Goodacre S, Cohen J, Bradburn M, Gray A, Benger J, Coats T. Intravenous or nebulised magnesium sulphate versus standard therapy for severe acute asthma (3Mg trial): a double-blind, randomised controlled trial. Lancet Respir Med 2013;1(4):293-300.

47.

Egelund TA, Wassil SK, Edwards EM, Linden S, Irazuzta JE. High-dose magnesium sulfate infusion protocol for status asthmaticus: a safety and pharmacokinetics cohort study. Intensive Care Med 2013;39(1):117-22.

48.

Irazuzta J, Egelund T, Wassil SK, Hampp C. Feasibility of short-term infusion of magnesium sulfate in pediatric patients with status asthmaticus. J Pediatr Pharmacol Ther 2012;17(2):150-4.

49.

Vaiyani D, Irazuzta JE. Comparison of Two High-Dose Magnesium Infusion Regimens in the Treatment of Status Asthmaticus. J Pediatr Pharmacol Ther 2016;21(3):233-8.

50.

Werner HA. Status asthmaticus in children: a review. Chest 2001;119(6):1913-29.

51.

Sarganas G, Schaffrath Rosario A, Berger S, Neuhauser HK. An unambiguous definition of pediatric hypotension is still lacking: Gaps between two percentile-based definitions

Version Date: June 25, 2025 (v3.0)

Page **51** of **54**


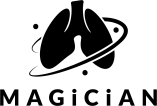


**MAGICIAN** Clinical Trial Protocol

and Pediatric Advanced Life Support/Advanced Trauma Life Support guidelines. J Trauma Acute Care Surg 2019;86(3):448-453.

52.

Trottier ED, Chan K, Allain D, Chauvin-Kimoff L. Managing an acute asthma exacerbation in children. Paediatr Child Health 2021;26(7):438-439.

53.

Chalut DS, Ducharme FM, Davis GM. The Preschool Respiratory Assessment Measure (PRAM): a responsive index of acute asthma severity. J Pediatr 2000;137(6):762-8.

54.

Birken CS, Parkin PC, Macarthur C. Asthma severity scores for preschoolers displayed weaknesses in reliability, validity, and responsiveness. J Clin Epidemiol 2004;57(11):1177-81.

55.

Panickar J, Lakhanpaul M, Lambert PC, et al. Oral prednisolone for preschool children with acute virus-induced wheezing. N Engl J Med 2009;360(4):329-38.

56.

Akinbami LJ, Schoendorf KC. Trends in childhood asthma: prevalence, health care utilization, and mortality. Pediatrics 2002;110(2 Pt 1):315-22.

57.

Lehr AR, McKinney ML, Gouin S, Blais JG, Pusic MV, Ducharme FM. Development and pretesting of an electronic learning module to train health care professionals on the use of the Pediatric Respiratory Assessment Measure to assess acute asthma severity. Can Respir J 2013;20(6):435-41.

58.

Kazi U, Rukh SG, Zawawi S, Laila S, Fareeduddin M, Saleem SG. To determine the association between asthma severity and hospital admission measured by Pediatric Respiratory Assessment Measure (PRAM) score at Indus Hospital and Health Network, Karachi, Pakistan, 2020-2021. Pak J Med Sci 2022;38(2):345-350.

59.

Yang CL, Hicks, E. A., Mitchell, P., Reisman, J., Podgers, D., Hayward, K. M., Waite, M., & Ramsey, C. D. 2021 Canadian Thoracic Society Guideline - A focused update on the management of very mild and mild asthma. Canadian Journal of Respiratory, Critical Care, and Sleep Medicine 2021;5(4):205–245.

60.

Becker SM, Job KM, Lima K, et al. Prospective study of serum and ionized magnesium pharmacokinetics in the treatment of children with severe acute asthma. Eur J Clin Pharmacol 2019;75(1):59-66.

61.

Qureshi F, Pestian J, Davis P, Zaritsky A. Effect of nebulized ipratropium on the hospitalization rates of children with asthma. N Engl J Med 1998;339(15):1030-5.

62.

Ducharme FM, Dell SD, Radhakrishnan D, et al. Diagnosis and management of asthma in preschoolers: A Canadian Thoracic Society and Canadian Paediatric Society position paper. Paediatr Child Health 2015;20(7):353-71.

63.

Jones BP, Paul A. Management of acute asthma in the pediatric patient: an evidence-

Version Date: June 25, 2025 (v3.0)

Page **52** of **54**


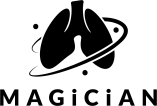


**MAGICIAN** Clinical Trial Protocol

based review. Pediatr Emerg Med Pract 2013;10(5):1-23; quiz 23-4.

64.

Craig SS, Dalziel SR, Powell CVE, Graudins A, Babl FE, Lunny C. Interventions for escalation of therapy for acute exacerbations of asthma in children: An overview of Cochrane reviews. Paediatr Respir Rev 2021;38:63-65.

65.

Alnaji F, Zemek R, Barrowman N, Plint A. PRAM score as predictor of pediatric asthma hospitalization. Acad Emerg Med 2014;21(8):872-8.

66.

Bekhof J, Reimink R, Brand PL. Systematic review: insufficient validation of clinical scores for the assessment of acute dyspnoea in wheezing children. Paediatr Respir Rev 2014;15(1):98-112.

67.

Eggink H, Brand P, Reimink R, Bekhof J. Clinical Scores for Dyspnoea Severity in Children: A Prospective Validation Study. PLoS One 2016;11(7):e0157724.

68.

Johnson MD, Nkoy FL, Sheng X, Greene T, Stone BL, Garvin J. Direct concurrent comparison of multiple pediatric acute asthma scoring instruments. J Asthma 2017;54(7):741-753.

69.

Arnold DH, Gebretsadik T, Hartert TV. Spirometry and PRAM severity score changes during pediatric acute asthma exacerbation treatment in a pediatric emergency department. J Asthma 2013;50(2):204-8.

70.

(CDC) CfDCaP. Asthma mortality and hospitalization among children and young adults-- United States, 1980-1993. MMWR Morb Mortal Wkly Rep 1996;45(17):350-3.

71.

Bjerg A, Sandström T, Lundbäck B, Rönmark E. Time trends in asthma and wheeze in Swedish children 1996-2006: prevalence and risk factors by sex. Allergy 2010;65(1):48- 55.

72.

Castro-Rodríguez JA, Holberg CJ, Wright AL, Martinez FD. A clinical index to define risk of asthma in young children with recurrent wheezing. Am J Respir Crit Care Med 2000;162(4 Pt 1):1403-6.

73.

Brand PL, Baraldi E, Bisgaard H, et al. Definition, assessment and treatment of wheezing disorders in preschool children: an evidence-based approach. Eur Respir J 2008;32(4):1096-110.

74.

Health CAfDaTi. Guidelines for the economic evaluation of health technologies: Canada. 2006.

75.

Schuh S, Coates AL, Dick P, et al. A single versus multiple doses of dexamethasone in infants wheezing for the first time. Pediatr Pulmonol 2008;43(9):844-50.

76.

Schuh S, Willan AR, Stephens D, Dick PT, Coates A. Can montelukast shorten

Version Date: June 25, 2025 (v3.0)

Page **53** of **54**


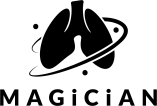


**MAGICIAN** Clinical Trial Protocol

prednisolone therapy in children with mild to moderate acute asthma? A randomized controlled trial. J Pediatr 2009;155(6):795-800.

77.

US Committee on Extension to the Standard Atmosphere. US Standard Atmosphere, 1976. US Government Printing Office; 1976.

Version Date: June 25, 2025 (v3.0)

Page **54** of **54**
