# Supplementary material for: Magnesium in acute pediatric asthma in the emergency department (MAGICIAN)—A multicentre randomized controlled clinical trial protocol
Source: PLoS One. 2026 Jun 3;21(6):e0349553. doi: 10.1371/journal.pone.0349553 (PMC13232952; doi:10.1371/journal.pone.0349553)
Supplement: S1 Appendix — (DOCX) [file pone.0349553.s003.docx]

# APPENDIX A - Abstract from our MAGNUM RCT

**Effect of Nebulized Magnesium vs Placebo Added to Albuterol on Hospitalization Among Children with Refractory Acute Asthma Treated in the Emergency Department-A Randomized Clinical Trial**

Suzanne Schuh, MD; Judy Sweeney, RN, BScN; Maggie Rumantir, MD; Allan L. Coates, MDCM, BEng; Andrew R. Willan, PhD; Derek Stephens, MSc, BSc; Eshetu G. Atenafu, MSc; Yaron Finkelstein, MD; Graham Thompson, MD; Roger Zemek, MD; Amy C. Plint, MD, MSc; Jocelyn Gravel, MD, MSc; Francine M. Ducharme, MD, MSc; David W. Johnson, MD; Karen Black, MD, MSc; Sarah Curtis, MD; Darcy Beer, MD; Terry P. Klassen, MD, MSc; Darcy Nicksy, BSc, PhM; Stephen B. Freedman, MDCM, MSc; for the Pediatric Emergency Research Canada (PERC) Network

***JAMA*. 2020**;324(20):2038-2047. doi:10.1001/jama.2020.19839

**Importance:** While intravenous magnesium decreases hospitalizations in refractory pediatric

acute asthma, it is variably used because of invasiveness and safety concerns. The benefit of

nebulized magnesium to prevent hospitalization is unknown.

**Objective:** To evaluate the effectiveness of nebulized magnesium in children with acute

asthma remaining in moderate or severe respiratory distress after initial therapy.

**Design, Setting and Participants:** A randomized double-blind parallel-group clinical trial

from September 26, 2011, to November 19, 2019, in 7 tertiary-care pediatric emergency

departments in Canada. The participants were otherwise healthy children aged 2 to 17 years

with moderate to severe asthma defined by a Pediatric Respiratory Assessment Measure

(PRAM) score of 5 or greater (on a 12-point scale) after a 1-hour treatment with an oral

corticosteroid and 3 inhaled albuterol and ipratropium treatments. Of 5846 screened

patients, 4332 were excluded for criteria, 273 declined participation, 423 otherwise excluded,

818 randomized, and 816 analyzed.

**Interventions:** Participants were randomized to 3 nebulized albuterol treatments with

either magnesium sulfate (n = 410) or 5.5%saline placebo (n = 408).

MAIN OUTCOMES AND MEASURES The primary outcome was hospitalization for asthma within

24 hours. Secondary outcomes included PRAM score; respiratory rate; oxygen saturation at

60, 120, 180, and 240 minutes; blood pressure at 20, 40, 60, 120, 180, and 240 minutes; and

albuterol treatments within 240 minutes.

**Results:** Among 818 randomized patients (median age, 5 years; 63%males), 816 completed

the trial (409 received magnesium; 407placebo). A total of 178 of the 409 children who

received magnesium (43.5%) were hospitalized vs 194 of the 407 who received placebo

(47.7%) (difference, −4.2%; absolute risk difference 95%[exact] CI, −11% to 2.8%]; *P* = .26).

There were no significant between-group differences in changes from baseline to 240

minutes in PRAM score (difference of changes, 0.14 points [95% CI, −0.23 to 0.50]; *P* = .46);

respiratory rate (0.17 breaths/min [95%CI, −1.32 to 1.67]; *P* = .82); oxygen saturation

(−0.04% [95%CI, −0.53%to 0.46%]; *P* = .88); systolic blood pressure (0.78mmHg [95%

CI, −1.48 to 3.03]; *P* = .50); or mean number of additional albuterol treatments (magnesium:

1.49, placebo: 1.59; risk ratio, 0.94 [95%CI, 0.79 to 1.11]; *P* = .47). Nausea/vomiting or sore

throat/nose occurred in 17 of the 409 children who received magnesium (4%) and 5 of the

407 who received placebo (1%).

**Conclusions and Relevance:** Among children with refractory acute asthma in the emergency department, nebulized magnesium with albuterol, compared with placebo with

albuterol did not significantly decrease the hospitalization rate for asthma within 24 hours.

The findings do not support use of nebulized magnesium with albuterol among children with

refractory acute asthma.

# APPENDIX B - Pediatric Respiratory Assessment Measure (PRAM)^a^ score

| **Signs** | **0** | **1** | **2** | **3** |
| --- | --- | --- | --- | --- |
| Suprasternal retractions | Absent |  | Present |  |
| Scalene muscle  contraction | Absent |  | Present |  |
| Air entry | Normal | Decreased at bases | Widespread decrease | Absent/minimal |
| Wheezing | Absent | Expiratory only | Inspiratory and  expiratory | Audible without  stethoscope/  silent chest with  minimal air entry |
| O2 saturation | ≥95% | 92%-94% | ≤91% |  |
| O2 saturation  Calgary^b^ | ≥93% | 90%-92% | ≤89% |  |

^a^ PRAM score represents the sum of the individual components (range 0-12).[25]

^b^ Because Calgary is 1000 meters above sea level, the saturation cut-offs have been adjusted accordingly.[77]

# APPENDIX C - Abstract from our secondary MAGNUM analysis

**Association between Intravenous Magnesium and Hospitalization from Emergency Departments in Pediatric Refractory Acute Asthma**

Suzanne Schuh, MD; Stephen B. Freedman, MDCM, MSc; Roger Zemek, MD; Amy C. Plint, MD, MSc; David W. Johnson, MD; Francine Ducharme, MD, MSc; Jocelyn Gravel, MD, MSc; Graham Thompson, MD; Sarah Curtis, MD; Derek Stephens, MSc, BSc; Allan L. Coates, MDCM, BEng; Karen J Black, MD, MSc; Darcy Beer, MD; Judy Sweeney, RN, BScN; Maggie Rumantir, MD; Yaron Finkelstein, MD; for the Pediatric Emergency Research Canada (PERC) Network

**JAMA Network Open, 2021**

**Importance:** Despite the guideline recommendation of IV magnesium to reduce hospitalizations in refractory pediatric acute asthma and increasing IV magnesium use, asthma-related hospitalizations have remained stable. Emergency department (ED) physicians may hesitate to discharge children home after IV magnesium.

**Objective:** To determine the association between IV magnesium therapy in the ED and hospitalization, after adjustment for patient-level variables. We hypothesized that children given IV magnesium would be at higher likelihood of hospitalization.

**Design:** Prospective cohort study representing a planned secondary analysis of a double-blind clinical trial of children with acute asthma randomized to three nebulized treatments of albuterol plus either magnesium sulfate or 5.5% saline placebo. Thereafter, IV magnesium therapy was used as per ED physician decision, representing the intervention of interest in the current study.

**Setting:** Seven Canadian tertiary-care pediatric EDs.

**Participants:** 816 otherwise healthy children 2-17 years old with Pediatric Respiratory Assessment Measure (PRAM) ≥5/12 points after initial therapy with systemic corticosteroids and inhaled albuterol with ipratropium.

**Exposure: IV magnesium sulfate (40-50 mg/kg) after inhaled magnesium/placebo.**

**Main Outcome(s) and Measure(s):**  Hospitalization for asthma from the ED. Candidate predictor variables included year-epoch at enrollment, IV magnesium, PRAM after initial therapy, PRAM at ED disposition, age, sex, respiratory distress duration, previous ICU asthma admission, asthma hospitalizations within the past year, atopy, oral corticosteroids within 48 hours before arrival, nebulized magnesium, and additional albuterol after inhaled magnesium/placebo, with site as a random effect.

**Results:** 364/816 (44.6%) patients were hospitalized, 215/816 (26.3%) received IV magnesium; 190/215 (88.4%) children given IV magnesium were hospitalized. Multivariable predictors of hospitalization were IV magnesium in 2011-2016 [OR 22.67(95% CI 6.26-82.06) p<0.001] and in 2017-2019 [OR 4.19 (1.99-8.86) p<0.001], additional albuterol [OR 5.53(3.27-9.37) p<0.001] and disposition PRAM [OR 2.28(1.92-2.71) p<0.001].

**Conclusions and Relevance:** After adjustment for patient-level characteristics, IV magnesium after initial ED asthma treatment is associated with hospitalization. Definitive evidence of IV magnesium benefit on hospitalization would clarify its role in refractory pediatric asthma.

# APPENDIX D - Abstract from our international PERN survey

**International Practice Patterns in Intravenous Magnesium Therapy in Pediatric Refractory Acute Asthma**

Laura Simone MD, MSc(c), FRCPC, Roger Zemek, MD; Damian Roland, MD, BMBS; Mark D Lyttle, MBChB; Simon Craig; Stuart R Dalziel, MBChB, FRACP, PhD; Jocelyn Gravel; Yaron Finkelstein, MD; Sarah Curtis, MD; Stephen B. Freedman, MDCM, MSc; Amy C. Plint , MD MSc; Naveen Poonai, MD, MSc; Franz E Babl, MD, MPH; Graham Thompson, MD, MSc; Francine M Ducharme, MD, MSc; David W. Johnson, MD; Terry P Klassen, MD, MSc; Bethany Lerman; Suzanne Schuh, MD for the Pediatric Emergency Research Canada (PERN) Network

**Emergency Medicine Journal, 2022**

**Background and Objectives**

Guidelines recommend intravenous magnesium (IV-Mg) in pediatric severe refractory asthma, despite limited evidence of benefit. The primary objective was to determine the proportion of physicians agreeing there is adequate evidence that children with sustained satisfactory response to IV-Mg in the emergency department can be safely discharged.

**Methods**

We conducted a cross-sectional survey of pediatric emergency physicians at research networks in Canada, Australia/New Zealand and the U.K./Ireland (March-June 2021), using a modified Dillman technique. Two clinical vignettes, describing a case of severe refractory asthma, preceded the survey. After bronchodilator and corticosteroid therapy, asthma severity in vignette 1 does not change and becomes moderate in vignette 2.

**Results**

657/886 (74%) physicians responded; 586 were eligible.104/586 (17.7%) participants stated that evidence regarding the safety of discharging patients with satisfactory response to IV-Mg is sufficient to support adoption. The goal of IV-Mg to decrease hospitalization in Canada vs elsewhere was expressed by 52.7% vs 3.3% participants in vignette 1 [OR 32.3 (17.1-64.7] and 51.9% vs 4.0% in vignette 2 [OR 50.4 (24.3-115.6]. A total of 443/586 (76%) physicans use IV-Mg to provide respiratory distress relief. 488 respondents (83.3%) stated that prescribing IV-Mg also prompts them to hospitalize, irrespective of clinical response. 455/586 participants (77.6%) agree that a definitive trial is needed to clarify IV-Mg benefit and 80% indicate that evidence of IV Mg benefit would change their practice.

**Conclusions**

A minority of physicians believe there is adequate evidence for IV-Mg benefit. International variability exists in physician acceptability to discharge following a sustained satisfactory response to IV-Mg. Conclusive evidence of IV-Mg benefit would inform the role of IV-Mg in refractory pediatric asthma and clarify the safety of discharge.

# APPENDIX E - Economic analysis

The cost-effectiveness of IVMg versus placebo to alleviate respiratory distress will be assessed from the perspective of the Canadian public health care system. To this end, trial-based cost-utility analysis (CUA) and cost-effectiveness analyses (CEA) will be conducted, using a decision-tree framework. First, CUA will be performed, in which costs and quality-adjusted life years (QALYs) associated with each treatment will be estimated, and the incremental cost per QALY gained will be determined. Second, a CEA will be used to assess the incremental cost per alleviating respiratory distress (i.e., achieving mild asthma status indicated by PRAM ≤ 3 points). Although the MCID for PRAM in the MAGICIAN trial is a change of at least 1 point, the decision problem will focus on the alleviation of respiratory distress (PRAM ≤ 3 points) as this signifies dischargable mild asthma. Both analyses will be conducted over a 72-hour horizon, given that all costs and benefits of IVMg treatment are expected to be experienced within 72 hours. In the CUA, the accumulated QALYs for both treatments are expected to be small owing to the 72-hour time frame; however, the cost-effectiveness of IVMg will be informed by the incremental difference in QALYs (as part of the incremental cost-utility ratio calculation). All analyses will adhere to the Guidelines for the Economic Evaluation of Health Technologies: Canada (CADTH 2017; 4^th^ edition).

Comparative effectiveness of IVMg versus placebo will be derived from the MAGICIAN trial. Treatment response (PRAM indicating mild asthma [≤3 points]; secondary outcome c) will be assessed at 180 minutes. Patient disposition (i.e., discharge or admission to hospital for asthma) during the index ED visit; secondary outcome a), resource use (secondary outcomes d, e, f), and adverse events will additionally be obtained from the MAGICIAN trial. To determine costs, a unit cost will be applied to each type of resource, and the total cost will be estimated as the weighted sum of resource use (the sum of the product of the number of each resource item and its unit cost). Costs associated with AEs will be based on the observed frequency of each AE in the MAGICIAN trial and costs associated with their management. The resources, type of measurement, and source of the cost data are described in the following table:

| **Resource item** | **Source of cost** |
| --- | --- |
| Drug acquisition | Publically available list price |
| Drug administration | Provincial fee schedule (Ministry of Health and Long-Term Care) |
| Hospitalization | Provincial ministry (Ontario Case Costing Initiative) |
| Physician consultation | Provincial fee schedule (Ministry of Health and Long-Term Care) |
| Emergency department visit | Provincial fee schedule (Ministry of Health and Long-Term Care) |

For the CUA, utilities will be incorporated for treatment response (mild asthma) or no treatment response (moderate-to-severe asthma) and disutilities will be included for adverse events (e.g., hypotension). Utilities and disutilities will be obtained from the literature via a systematic search, with the choice of values based on their fitness for purpose, credibility, and consistency.

Probabilistic analyses will be used to incorporate uncertainty around the estimate of costs and outcomes. Scenario and sensitivity analyses will be undertaken to explore the impact of uncertainty within the analyses (e.g., structural or parameter uncertainty).

# APPENDIX F - Annual Enrollment Projection

|  | **Annual ED Asthma Presentations**  **≥2 years old** | **Projected**  **Annual**  **Screens+** | **Randomizations*** | **Projected Annual Study Numbers with Full Data **** |
| --- | --- | --- | --- | --- |
| **HSC** | 900 | 300 | 50 | 45 |
| **CHEO** | 900 | 200 | 14 | 12 |
| **Alberta Children’s** | 750 | 200 | 33 | 30 |
| **Stollery** | 300 | 100 | 6 | 5 |
| **St Justine** | 750 | 200 | 14 | 12 |
| **McMaster** | 300 | 100 | 6 | 5 |
|  |  |  |  |  |
| **Total** | **3900** | **1100** | **123** | **109** |

**+** Informed by MAGNUM schedule of 12 hours/day weekdays and 12 hours 1 weekend day a week; 10% miss rate, screens represent approximately 30% of annual presentations

***** Projected randomizations are conservatively estimated on 75% of screened children excluded for criteria, and approx 50% of eligible patients not participating due to refusals/non-participation for other reasons (based on MAGNUM).

****** Numbers with full data are conservatively based on 5% rate of non-compliance with allocated therapy and 1% loss to follow-up (1/818 in MAGNUM).
